# Pressure‐Induced Metallization and Isostructural Transitions in 3R‐MoS_2_

**DOI:** 10.1002/advs.202505031

**Published:** 2025-06-27

**Authors:** Azkar Saeed Ahmad, Mangladeep Bhullar, Kenny Stahl, Wenting Lu, Taiyi Chen, Lei Feng, Xin Hu, Qian Zhang, Konstantin Glazyrin, Martin Kunz, Yusheng Zhao, Shanmin Wang, Yansun Yao, Elissaios Stavrou

**Affiliations:** ^1^ Materials Science and Engineering Department Guangdong Technion‐Israel Institute of Technology Shantou 515063 China; ^2^ Guangdong Provincial Key Laboratory of Materials and Technologies for Energy Conversion (MATEC) Guangdong Technion‐Israel Institute of Technology Shantou 515063 China; ^3^ Department of Physics and Engineering Physics University of Saskatchewan Saskatoon Saskatchewan S7N 5E2 Canada; ^4^ Department of Chemistry Technical University of Denmark Lyngby DK‐2800 Denmark; ^5^ Deutsches Elektronen‐Synchrotron (DESY) D‐22603 Hamburg Germany; ^6^ Advanced Light Source Lawrence Berkeley Laboratory Berkeley CA 94720 USA; ^7^ Department of Physics Southern University of Science and Technology Shenzhen 518055 China; ^8^ Department of Materials Science and Engineering Technion‐Israel Institute of Technology Haifa 32000 Israel

**Keywords:** high pressure‐temperature synthesis, isostructural transitions, metallization, transition metal dichalcogenides, van der Waals forces

## Abstract

At ambient conditions 3R‐polytypes of transition metal dichalcogenides (TMDs) demonstrate fascinating properties because of their unique layer stacking. Understanding the structure‐property relationship is essential for the realization of their use in spintronic, valleytronic, and optoelectronic applications. Herein, after the high pressure‐temperature synthesis of 3R‐MoS_2_ in a large volume cubic press, a concomitant experimental and theoretical high‐pressure study of 3R‐MoS_2_ is reported, leading to the discovery of pressure‐induced reversible isostructural phase transitions without symmetry breaking. Concurrent with the isostructural transitions, a semiconductor‐to‐metal transition is observed, owing to strong interlayer interaction and charge redistribution across the van der Waals gap under pressure. The pressure‐induced enhancement of interlayer interactions together with the robust intrinsic layer stacking in 3R‐MoS_2_ prevent the layers from sliding under pressure and hinder a corresponding volume collapse. This study on continuous pressure‐tuning of crystal and electronic structure in 3R‐MoS_2_ will play a vital role in developing the next‐generation devices involving coupling of structural, optical, and electrical properties of 3R‐polytypes of TMDs and other layered materials.

## Graphical abstract

Pressure‐induced isostructural transitions without symmetry breaking are reported in 3R‐MoS_2_. Concurrently, a semiconductor‐to‐metal transition is observed, owing to strong interlayer interaction and charge redistribution across the van der Waals gap under pressure. The pressure‐induced enhancement of interlayer interactions together with the robust intrinsic layer stacking in 3R‐MoS_2_ prevent the layers from sliding and hinder a corresponding volume collapse.

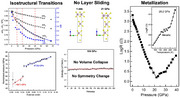

## 1 Introduction

Transition metal dichalcogenides (TMDs), including MoS_2_, have attracted great attention of the scientific community, being superior candidate materials for a variety of applications such as electrochemical catalysis,^[^ ^1^, ^2^ ^]^ sensing,^[^ ^3^, ^4^, ^5^ ^]^ energy storage and conversion.^[^ ^6^ ^]^ The increasing interest in exploring the origin of diverse properties and thus unique applications of TMDs stems from their layered structure, that is akin to that of graphite.^[^ ^7^, ^8^, ^9^ ^]^ MoS_2_, like other TMDs (e.g., WS_2_, MoSe_2_, etc.), exists as various polytypes, of which 2H, 3R, and 1T have been the most commonly known and extensively studied. Structurally, these TMDs are composed of MoS_2_ layers that are held together by weak van der Waals (vdW) forces and each layer is composed of covalently bonded condensed MoS_6_ centers, in which alternating sheets of sulfur atoms sandwich a sheet of molybdenum atoms. The basic structural difference between polytypes is in the shape of the MoS_6_ center (octahedral or trigonal prismatic) and the arrangement of the layers. In the case of 2H‐MoS_2_, which is the most thermodynamically stable structure, the arrangement of layers can be described by a hexagonal unit cell, with trigonal prismatic MoS_6_ centers in space group P63/mmc (No. 194),^[^ ^10^ ^]^ but other metastable polytypes are possible.^[^ ^11^ ^]^

Of these, the rhombohedral (3R‐MoS_2_) polytype in space group *R3m* (SG: 160)^[^ ^10^, ^12^ ^]^ is of primary interest as it is the second most stable polytype of MoS_2_ and it can be synthesized by subjecting 2H‐MoS_2_ to high pressure and temperature conditions.^[^ ^13^ ^]^ 3R‐MoS_2_ adopts a ABC‐ABC layer stacking order, as opposed to the AB‐AB layer stacking order of 2H‐MoS_2,_ and retains its broken inversion symmetry from monolayer to multilayer (bulk) exhibiting strong valley and spin polarization that are not observed in its 2H‐MoS_2_ counterpart.^[^ ^14^, ^15^ ^]^ Additionally, it has a tunable bandgap,^[^ ^16^ ^]^ high piezoelectric constant,^[^ ^17^ ^]^ and outperforms 2H‐MoS_2_ in hydrogen evolution reactions.^[^ ^18^ ^]^ These traits make it a promising material for engineering,^[^ ^19^, ^20^ ^]^ catalysis,^[^ ^21^ ^]^ mineral processing,^[^ ^22^ ^]^ and photonic and optoelectronic devices.^[^ ^15^, ^17^ ^]^

Several methods have been utilized to tune/alter the properties of the 2H‐MoS_2,_ aiming for its use in next‐generation photonics and optoelectronics, including defect engineering,^[^ ^23^, ^24^, ^25^ ^]^ intercalation,^[^ ^26^, ^27^ ^]^ chemical doping,^[^ ^25^, ^28^, ^29^, ^30^ ^]^ and surface functionalization.^[^ ^31^ ^]^ Recently, pressure has been opted as a powerful tool for continuously tuning the structural and electronic properties of 2H‐MoS_2_ away from the pristine states. 2H‐MoS_2_ has been found to demonstrate pressure‐induced metallization associated with structural transitions.^[^ ^32^, ^33^ ^]^ On the other hand, 3R‐MoS_2,_ despite its great technological importance, has not yet been explored under pressure. In this article, combining our experimental and theoretical studies, we explore the high‐pressure behavior of 3R‐MoS_2,_ and report pressure‐induced isostructural transitions and metallization in 3R‐MoS_2_. In addition, we discuss our results on metallization due to bandgap closuring with reference to previous reports on bandgap tuning and enhanced interlayer interactions due to electrostatic gating in 2H‐polytypes of TMDs.^[^ ^16^, ^34^ ^]^ Our results present an analogy between electrostatic gating and pressure tuning of the bandgap and enhanced interlayer interactions. Such an analogy in the two different techniques manifests the use of pressure in structure and bandgap tuning for the next generation photonic and optoelectronic devices.

## 2 Results and Discussion

### 2.1 Synthesis

**Figure 1:**
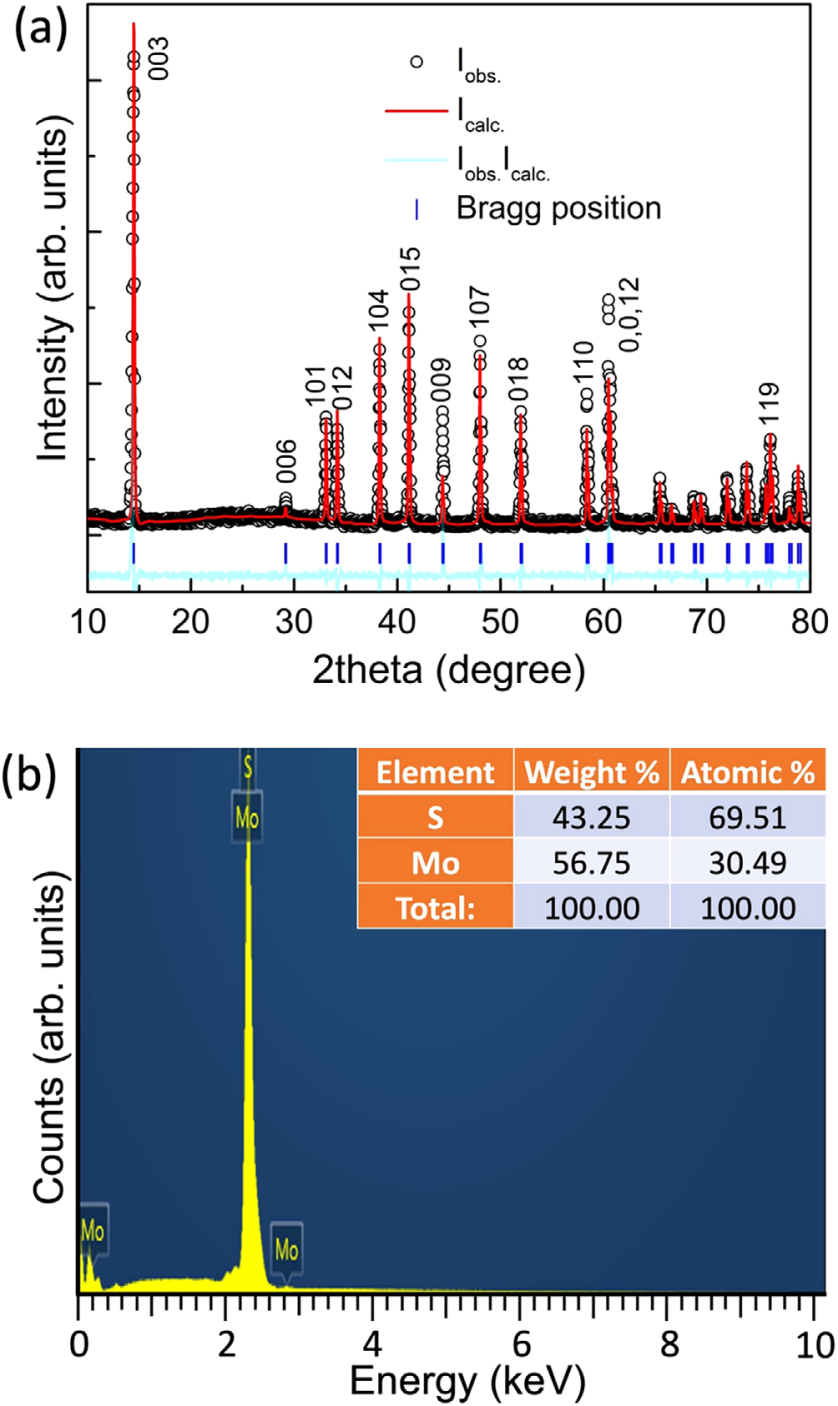
High pressure‐temperature synthesis and purity of 3R‐MoS_2_. a) X‐ray diffraction pattern of as‐synthesized 3R‐MoS_2_, collected at ambient conditions with a copper target. b) EDX spectra and elemental composition of as‐synthesized 3R‐MoS_2_.

3R‐MoS_2_ was synthesized through a solid‐state high pressure‐temperature reaction in a large‐volume cubic press by subjecting 2H‐MoS_2_ to a pressure of 5 GPa and a temperature of 1800 °C for 15 min. The specimen was then quenched and the synthesis was confirmed by x‐ray diffraction (XRD) and energy dispersive x‐ray (EDX) analysis (**Figure** **1**). The structural parameters of as‐synthesized 3R‐MoS_2_ at ambient conditions are consistent with the previous reports^[^ ^13^, ^35^ ^]^ and are listed in the Table S1 (Supporting Information).

### 2.2 Synchrotron XRD

**Figure 2:**
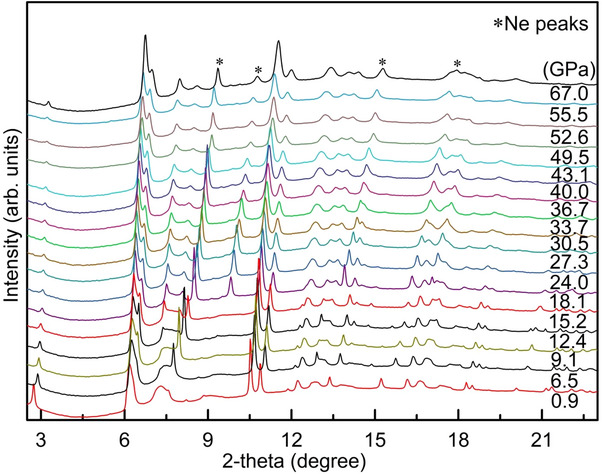
Representative synchrotron XRD patterns during compression. Diffraction peaks from the pressure transmitting medium (i.e., neon) have been marked with asterisks.

**Figure 3:**
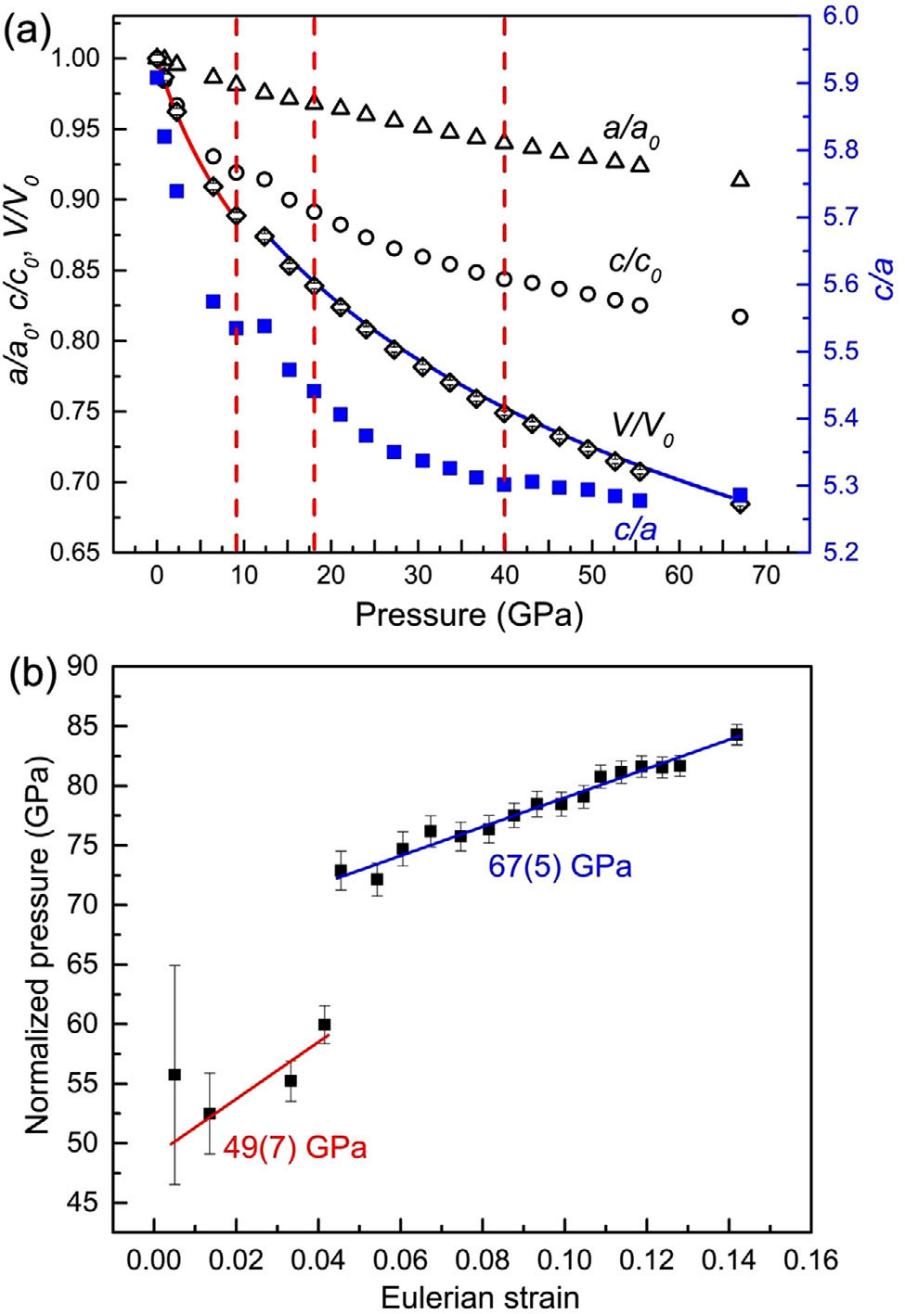
Structural lattice parameters of 3R‐MoS_2_. a) Experimental pressure‐dependence of normalized lattice parameters and volume of 3R‐MoS_2_. The small volume uncertainties are indicated with the bars within the symbol size. Solid lines are the fitted curve using a 3rd order Birch‐Munaghan equation of state. Vertical dashed lines mark the transition pressures. b) Normalized pressure (*F*) vs Eulerian strain (*f*) plot.

XRD patterns of 3R‐MoS_2_ at selected pressures at 300K are displayed in **Figure** **2**. We note that some peaks (e.g., peaks ≈6.2° and 7.3°) are asymmetrically broadened, and at the same time, the intensities of the peaks increase gradually with increasing pressure which can be attributed to preferred orientation in multilayered 3R‐MoS_2_. Similar pressure‐dependence of the peaks shape has also been observed in other layered materials.^[^ ^41^, ^42^, ^43^ ^]^ Up to 67 GPa, all XRD patterns can be indexed with the space group *R3m* phase without signs of symmetry changes (Figure S1, Supporting Information). However, apparent discontinuities are observed in the plots of the volume (*V/V_0_*) and axis ratio *c/a* against pressure (**Figure** **3**a), suggesting lattice distortions in the local structure and hence the isostructural transitions at pressures of ≈9.1, ≈18.0, and ≈40.0 GPa. The obtained pressure–volume (PV) data can be described by two independent third‐order Birch–Murnaghan equation of states (EoS), giving bulk moduli (*K*_0_) of 49(7) and 67(5) GPa with their first pressure derivatives ($K_{0}^{\prime }$) of 7.3(2.6) and 5.2(0.2) for the low pressure (low‐P) and high pressure (high‐P) phases, respectively. In the low‐P phase regime (1 atm to 9.1 GPa) a relatively sharp decrease of the c/*a* ratio originates from the fact that the *c*‐axis is more compressible under pressure, than the *a*‐axis, indicating a decrease of interlayer distance and an enhancement of the interlayer interactions.

The isostructural transition at ≈9.1 GPa is further confirmed by the normalized pressure versus Eulerian strain (*F‐f*) plot. As evident from Figure 3b, a change in the slope of *F*‐*f* plot indicates a discontinuity in *K*_0_ and/or $K_{0}^{\prime }$, and hence provides additional evidence that 3R‐MoS_2_ undergoes an isostructural phase transition at ≈9.1 GPa. It is worth noting that unlike to 2H‐MoS_2_, the 3R‐MoS_2_ does not exhibit any new diffraction feature at lower diffraction angles (Figure 2; Figures S1–S3, Supporting Information). The appearance of new diffraction feature at lower angles (i.e., higher d‐spacings) in 2H‐MoS_2_ was reported to be associated with the layer sliding and the corresponding volume collapse during the transformation to the new high‐P phase (i.e., 2H_a_‐phase). To further verify the absence of new low‐angle peaks in the case of 3R‐MoS_2_, we reperformed a second synchrotron XRD measurements run up to ≈57.4 GPa, and yet again, we do not observe any new diffraction features (Figure S3, Supporting Information). This documents the absence of the appearance of new diffraction features in 3R‐MoS_2_ under pressure, and consequently rules‐out the possibility of  layer sliding and corresponding volume collapse in the case of 3R‐MoS_2_. In other words, the high‐pressure behavior of 3R‐MoS_2_ strikingly differs from 2H‐MoS_2_.^[^ ^33^ ^]^ Upon complete decompression, all the isostructural transitions in 3R‐MoS_2_ are fully reversible (Figure S4, Supporting Information).

### 2.3 Raman Spectroscopy

**Figure 4:**
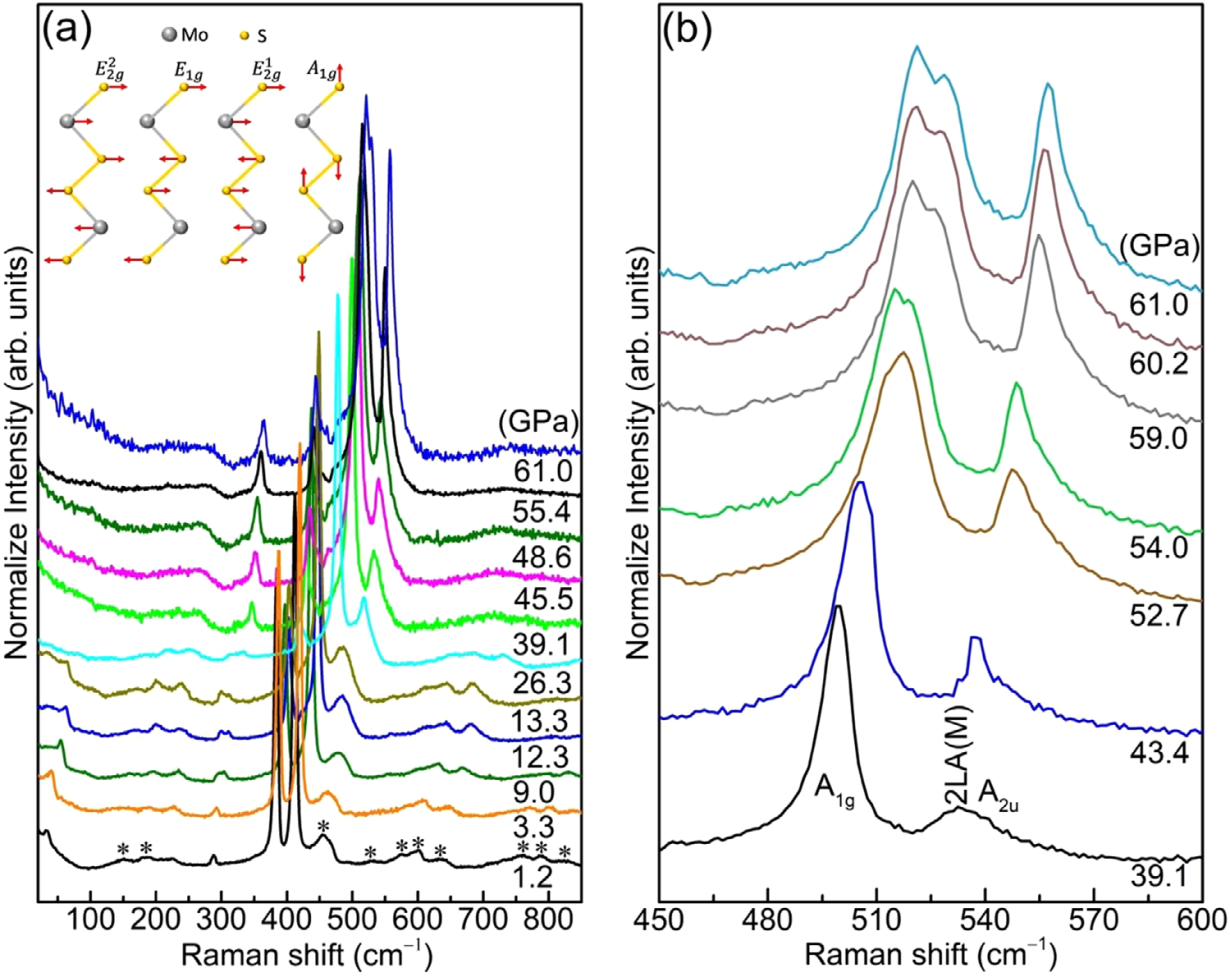
a) Raman spectra at selected pressures. The inset illustrates the vibrations of the Raman active modes b) Zoom in view of the *A*_1*g*_ and 2*LA*(*M*)/*A*_2*u*_ modes at selected pressures. Asterisks (∗) denote the low intensity and 2nd order modes and have been elaborated in Figure S5 (Supporting Information).

High‐pressure Raman spectroscopic measurements have been performed to reveal the isostructural phase transition in 3R‐MoS_2_. It is evident from **Figure** **4** and Figures S5 and S6 (Supporting Information) that around ambient conditions spectrum of 3R‐MoS_2_ exhibits four Raman active modes corresponding to the in‐plane $E_{2g}^{2}$, *E*_1*g*_, and $E_{2g}^{1}$, and the out‐of‐plane *A*_1*g*_ phonon modes.^[^ ^11^, ^44^, ^45^ ^]^ In addition to these Raman active modes, we also observe low intensity, 2nd order modes marked with asterisks in Figure 4 and elaborated in Figure S5 (Supporting Information). The $E_{2g}^{1}$ and $E_{2g}^{2}$ modes originate from the vibrations of Mo and S atoms in the basal plane, with the opposite directions within the MoS_2_ unit for $E_{2g}^{1}$ and the same direction within the MoS_2_ unit for $E_{2g}^{2}$. In the case of $E_{2g}^{2}$, all three atoms within a MoS_2_ unit move in the opposite direction of the neighboring MoS_2_. $E_{2g}^{2}$ is a rigid layer mode and its low frequency reflects the weak vdW interactions. The *E*_1*g*_ mode is from the vibration of only S atoms in the basal plane whereas, *A*_1*g*_ mode results from their vibrations along the *c*‐axis.

**Figure 5:**
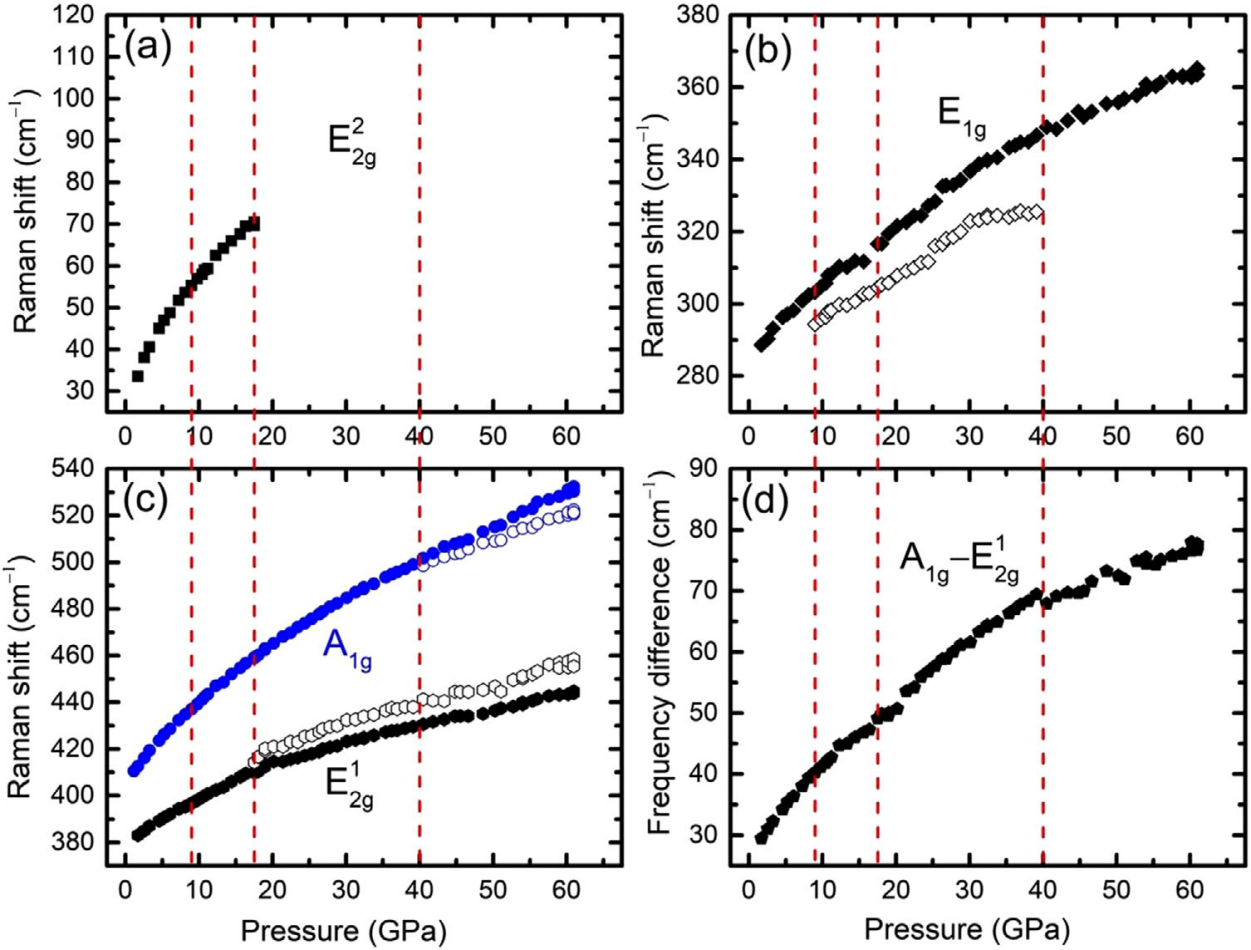
Phonon modes of 3R‐MoS_2_ as a function of pressure. Raman shift (frequency) as a function of pressure during compression in the spectral regions of in‐plane $E_{2g}^{2}$ a), *E*_1*g*_ b), and $E_{2g}^{1}$ modes and the out‐of‐plane *A*_1*g*_ mode c) and the frequency differences between *A*_1*g*_ and $E_{2g}^{1}$ d). Vertical dashed lines mark the transition pressures.

Upon compression, all four Raman active modes demonstrate distinct features. The plot of mode frequency versus pressure can be divided into four regions as shown in **Figure** **5**. The rigid layer mode $E_{2g}^{2}$, which corresponds to the weak vdW interlayer interactions, is found to be very sensitive under pressure as it moves toward higher frequency sharply upon compression up to ≈9.0 GPa indicating the enhancement of interlayer interaction and vdW forces. This corresponds well to the initial sharp drop in the lattice parameter *c* (Figure 3a). At the same pressure point, the splitting of *E*_1*g*_ mode indicates the 1st isostructural transition (Figure 4; Figures S6 and S7a, Supporting Information). With further pressure increase, the intensity of the $E_{2g}^{2}$ mode is diminished at ≈18.0 GPa, which contrasts with the behavior of the $E_{2g}^{2}$ mode in 2H‐MoS_2_ under pressure.^[^ ^33^ ^]^ At the same pressure of ≈18.0 GPa $E_{2g}^{1}$ mode develops a split off, supporting the onset of a 2nd isostructural transition in 3R‐MoS_2_. Upon further compression, the new mode that appeared because of the splitting in *E*_1*g*_ mode, finally disappears at ≈40 GPa. Around the same pressure value, unlike 2H‐MoS_2_, the *A*_1*g*_ mode in 3R‐MoS_2_ develops a split off (Figure 3) and a discontinuity is observed in the separation between *A*_1*g*_ and $E_{2g}^{1}$ (Figure 5d). Alongside, around the same pressure, a significant increase in the intensity of the broad envelope of the 2LA(M)/A_2u_ mode, indicates that this mode has become Raman active (Figure 4a,b; Figures S5 and S6, Supporting Information). Such a scenario has never been observed in other polytypes of TMDs (i.e., 2H‐MoS_2_).^[^ ^32^, ^33^ ^]^ In addition to the increase in intensity, the FWHM of this envelope substantially and monotonically decreases upon compression (Figure S8, Supporting Information). Generally, upon compression, the intensity of a Raman active mode is expected to decrease and the FWHM to increase. However, observing the inverse phenomenon in 3R‐MoS_2_ suggests that the A_2u_ mode becomes Raman active. In other words, this is in favor of the scenario where the isostructural transition makes the A_2u_ mode Raman active in the HP phase of 3R‐MoS_2_, instead of an 2LA mode. These observations altogether indicate the occurrence of a 3rd isostructural transition, in agreement with the XRD results (Figure 3a). Upon decompression, the isostructural phase transitions are again found to be reversible in Raman spectroscopy measurements (Figure S9, Supporting Information).

Although different pressure‐transmitting mediums (PTMs), i.e., Ne and He were used in our XRD and Raman spectroscopic measurements, respectively, the onset pressures corresponding to each isostructural transition are practically identical. Additionally, the observed isostructural transitions are found to be completely reversible from both XRD and Raman spectroscopic measurements. These observations of reproducibly of isostructural transitions and their reversibility in different experimental techniques employing different PTMs, rules‐out the possibility of any effects originating from the use of different PTMs and sample degradation due to non‐hydrostatic stresses.

### 2.4 Metadynamics Simulations

**Figure 6:**
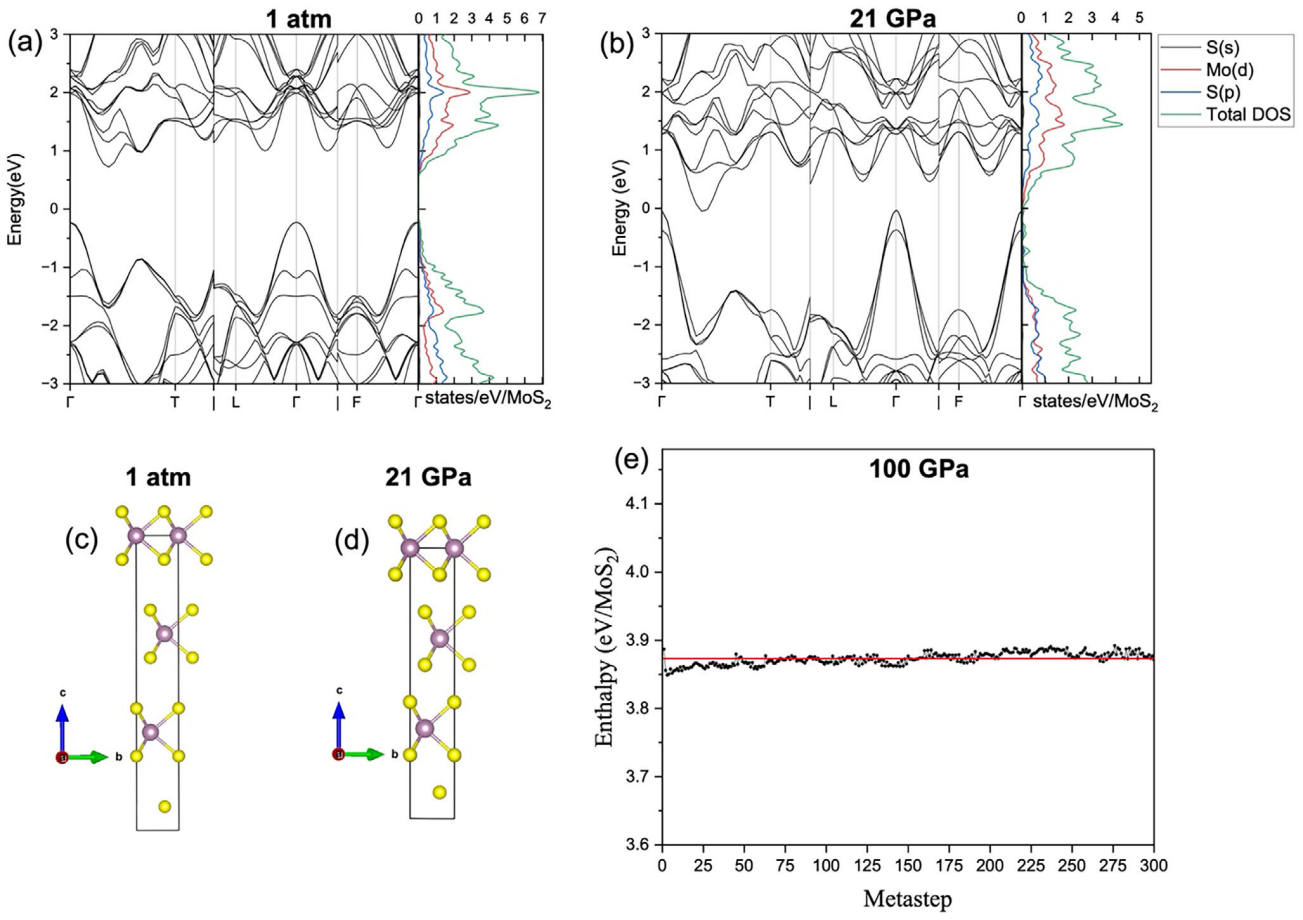
High pressure theoretical calculations of 3R‐MoS_2_. Band structure and density of states (DOS) at 1atm a) and 21 GPa b) and corresponding optimized structures at 0 GPa c), and 21 GPa d) using PBEsol functional. Big purple spheres represent Mo atoms and small yellow spheres represent S atoms. e) Evolution of enthalpy during metadynamics simulation at 100 GPa.

As described in the introduction, at ambient conditions 3R‐MoS_2_ adopts the ABC‐ABC layer stacking order as opposed to the AB‐AB stacking order of 2H‐MoS_2_ (2H_c_‐MoS_2_). Under high pressure conditions 2H_c_‐MoS_2_ undergoes layer sliding and corresponding volume collapse, and transforms to the 2H_a_‐MoS_2_, as reported by Chi et al.^[^ ^33^ ^]^ On the other hand, 3R‐MoS_2_ doesn't demonstrate such a pressure‐induced layer sliding and corresponding volume collapse. In simple words, in 3R‐MoS_2_, the ABC‐ABC layer stacking order remains intact under pressure. In order to further examine the absence of layer sliding in 3R‐MoS_2_ under pressure, we performed ab initio metadynamics simulations to predict the structural evolution of 3R‐MoS_2_ at 80 and 100 GPa, i.e. far above the highest pressures achieved in the experimental study. Metadynamics simulations at 80 and 100 GPa were conducted in a 3R‐MoS_2_ supercell of 72 atoms to investigate the role of interlayer interactions within the structure. Consistent with experimental results, the simulations show no change in the enthalpy, indicating the absence of any phase transition (sliding events) between the consecutive layers of 3R‐MoS_2_ structure, confirming the stability of the layers within the polytype. At 100 GPa, the enthalpy fluctuates within a narrow range of 3.848 and 3.892 eV, with an average of 3.873 eV highlighted by the red line in **Figure** **6**e. Metadynamics simulations at 80 GPa yielded the same conclusion. Thus, theory does not predict any first‐order phase transition for 3R‐MoS_2_, up to at least 100 GPa, while the observed isostructural transitions represent marginal enthalpy differences inaccessible by theoretical calculations. The lack of sliding events points to a strong coupling present in the 3R‐MoS_2_ polytype, which hinders any layer displacements/slidings and thus rules out any first‐order phase transitions in this polytypic structure, in striking contrast with the case of 2H‐MoS_2_.^[^ ^33^, ^46^ ^]^ The absence of layer sliding means that the ABC‐ABC layer stacking order, which is responsible for broken inversion symmetry, is persevered under pressure, making 3R‐MoS_2_ a candidate material for the extreme condition nonlinear photonic and valleytronic devices.

### 2.5 Electrical Measurements

**Figure 7:**
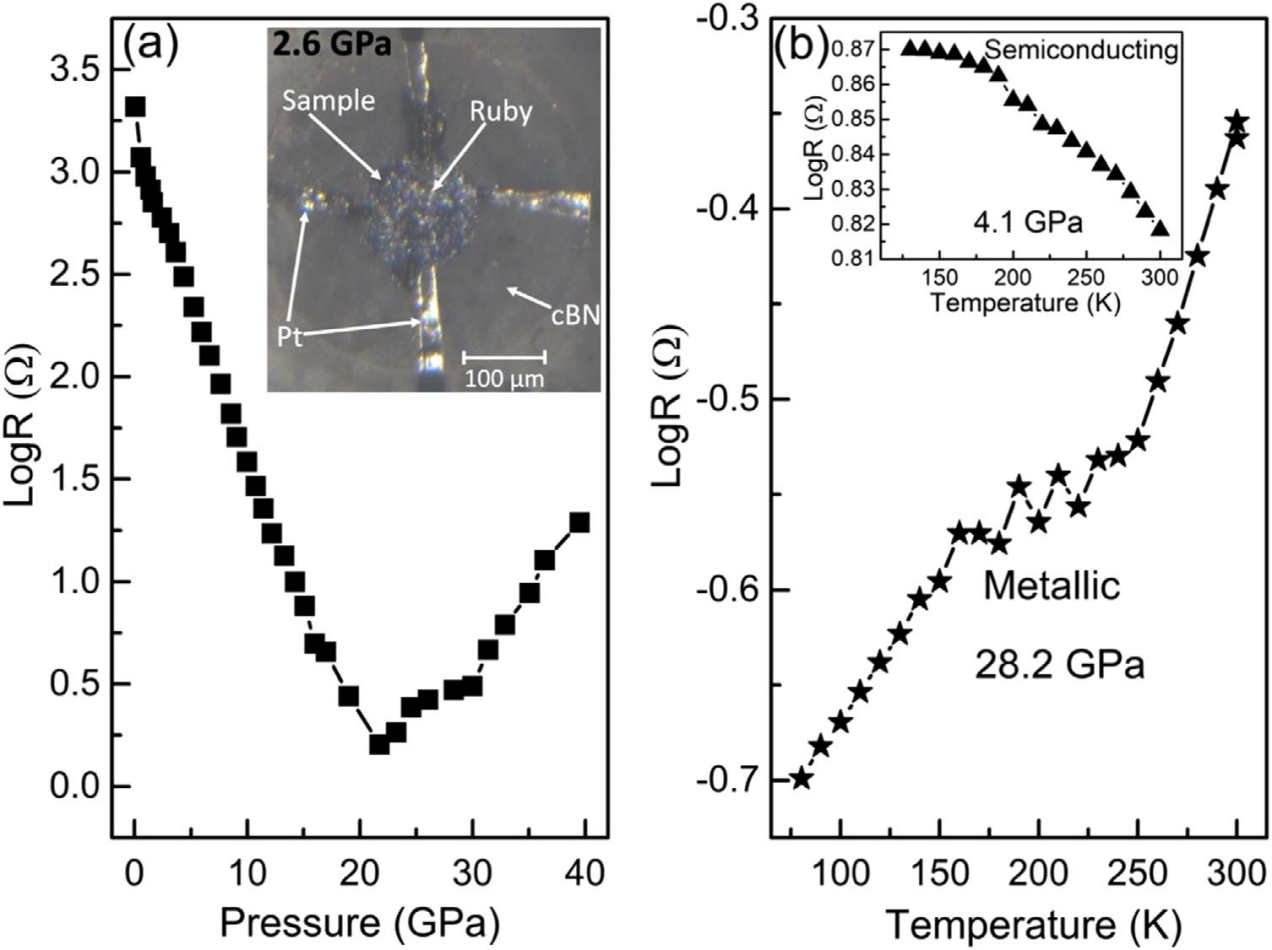
High pressure electrical resistance measurements. Pressure‐dependence of the room temperature electrical resistance of 3R‐MoS_2_ a). Inset of (a) is the photograph of the setup used for the electrical measurements illustrating the sample (3R‐MoS_2_) inside the diamond anvil cell together with ruby, cBN and Pt leads at 2.6 GPa. Temperature‐dependence of the electrical resistance at 28.2 GPa when 3R‐MoS_2_ is in metallic state b). Inset of (b) is the temperature‐dependence of electrical resistance at 4.1 GPa when 3R‐MoS_2_ is in semiconducting state.

To investigate the electronic response to pressure, we performed in situ high‐pressure electrical resistance measurements on 3R‐MoS_2_ up to ≈40.0 GPa (**Figure** **7**). As the pressure increases, the electrical resistance is found to decrease sharply up to the pressure of ≈18.0 GPa, and this can be related to the metallization of 3R‐MoS_2_. This sharp drop in the resistance of 3R‐MoS_2_ significantly differs from the two‐step resistivity drop in 2H‐MoS_2_.^[^ ^32^, ^33^ ^]^ Another striking difference is that the metallization in 3R‐MoS_2_ occurs in the same phase, whereas, in case of 2H‐MoS_2_ it occurs in a new high‐pressure phase (i.e., 2H_a_‐phase).^[^ ^33^ ^]^ It should be noted that there is a relatively sharp rise in electrical resistance of 3R‐MoS_2_ after 20 GPa, which can be attributed to strong electron scattering from grain boundaries in powder samples and pressure‐induced disorders/defects under non‐hydrostatic pressure conditions. To confirm the pressure‐induced metallization in 3R‐MoS_2_, four‐probe temperature‐dependent resistance measurements were performed. Classical semiconducting (Figure 7b (inset) and Figure S10, Supporting Information) and metallic (Figure 7b) behaviors are reflected at low (i.e., 4.1 and 14.0 GPa) and high (i.e., 28.2 GPa) pressures, respectively. It is also interesting to note that the resistance versus temperature curve exhibits an irregular shape between 170 to 240K (Figure 7b). This was also observed in 2H‐MoS_2_ and suggested to be associated with charge‐density wave.^[^ ^33^ ^]^

As described above, our electrical resistance measurements confirm the pressure‐induced semiconductor‐to‐metal transition in 3R‐MoS₂. However, to gain a deeper insight into the exact mechanism of the bandgap closure and the role of doping levels during metallization, synchrotron‐based far‐infrared (FIR) spectroscopy measurements are crucial. Such measurements have previously been performed on several 2H‐polytypes (e.g., MoS₂, WS₂, and MoTe₂),^[^ ^34^, ^47^ ^]^ aiming to not only resolve the controversy regarding the onset metallization pressure but also elucidate the role of doping levels during metallization process. In these studies, the experimental results were interpreted within the framework of Fano theory,^[^ ^48^ ^]^ revealing a general trend in 2H‐polytypes: hybridization between doping levels and conduction band states results to early metallization, whereas conventional bandgap closure occurs at relatively higher pressures. The above‐mentioned previous reports of nearly identical phenomena in three distinct 2H‐polype TMDs (i.e. MoS₂, WS₂, and MoTe₂),^[^ ^34^, ^47^ ^]^ despite the naturally expected different onset metallization pressures, document that doping levels play a vital role in the pressure‐tuning of the electronic properties of most of the TMDs. Therefore, the role of doping levels shall be taken into account while studying the pressure‐induced metallization in TMDs. Given the strikingly different high‐pressure structural behavior of 3R‐MoS₂ observed in this study compared to previous reports on the 2H‐polytype,^[^ ^32^, ^33^ ^]^ it would be worthwhile to perform high‐pressure FIR measurements on 3R‐MoS₂ in future studies, to uncover possible new insights into the mechanisms driving pressure‐induced metallization. Such a study on 3R‐MoS₂, utilizing FIR measurements and interpreting the results in reference to the previous models on 2H‐polytypes,^[^ ^34^, ^47^ ^, 49]^ would elucidate whether the role of doping levels in pressure‐induced metallization is a common feature across all types of TMDs (i.e., both 2H‐ and 3R‐polytpes).

### 2.6 Ab Initio Calculations

To better understand the electronic structure evolution and the corresponding pressure‐induced metallization in 3R‐MoS_2_, ab initio calculations were performed using PBEsol (Figure 6) and optPBE‐vdW functionals (Figures S11 and S12 and Table S2, Supporting Information). At ambient conditions (1 atm), 3R‐MoS_2_ has an indirect bandgap of ≈1.0 eV from both PBEsol and optPBE‐vdW functionals, consistent with the previous reports.^[^ ^11^, ^50^ ^]^ Upon compression, when using PBEsol functional, metallization is predicted at 21.0 GPa (Figure 6b), in well agreement with the experimental findings (Figure 7). However, when the calculations were performed using optPBE‐vdW, metallization is predicted at a relatively higher pressure of 31.5 GPa (Figure S11, Supporting Information). The difference in the onset of metallization pressure in two functionals (PBEsol and optPBE‐vdW) can be understood by considering the fact that vdW interactions are less prominent under pressure. It is worth noting that for both PBEsol and optPBE‐vdW functionals, the metallization and indirect nature of the bandgap is well preserved up to the maximum pressure achieved (Figure 6b; Figure S11 and Table S2, Supporting Information). Therefore, it can be concluded that while performing high‐pressure bandgap calculations on TMDS, PBEsol functional can be preferred as it corresponds well to the experimental findings. In addition to electronic structure, we also examined the atomic structure evolution under pressure with a specific focus on layer stacking ordering. It is obvious that ABC‐ABC layer stacking order in 3R‐MoS_2_ remains intact without undergoing any slippage in both methods employing PBEsol (Figure 6c,d) and optPBE‐vdW (Figure S12, Supporting Information) functionals. Thus, our theoretical calculations correspond well to our experiments, ruling out the possibility of corresponding volume collapse and first order phase transition in 3R‐MoS_2_ as opposed to 2H‐MoS_2_.^[^ ^33^ ^]^

To explore the orbital involvement in the charge redistribution and corresponding metallization, we performed angular momentum projected density of states (PDOS) of Mo and S in multilayered 3R‐MoS_2_ at two selected pressures (Figure S13, Supporting Information). It is obvious that both conduction band (CB) and valence band (VB) are mainly originated from the S‐*p* and Mo‐*d* orbitals. Importantly, at ambient conditions, the conduction band minimum (CBM) and valence band maximum (VBM) mainly result from the S‐*p_y_*, S‐*p_z_*, S‐*p_x_*, and Mo‐$d_{x^{2}-y^{2}}$, Mo‐$d_{z^{2}}$, Mo‐*d_xy_* orbitals. Upon compression to 21.0 GPa, the bandgap closes, and the specimen becomes metallic (Figure 6b), and the contribution to VBM and CBM from S‐*p_y_* and S‐*p_x_* orbitals decreases, whereas the contribution from the S‐*p_z_* and Mo‐$d_{z^{2}}$ orbitals increases (Figure S13, Supporting Information), indicating charge redistribution and greatly enhanced interlayer interactions. Such an increase in interlayer interactions is corroborated by our XRD and Raman spectroscopy results. In particular, the rapid drop in lattice parameter *c* (which corresponds to the interlayer spacing, Figure 3) and the splitting of *A*_1*g*_ mode (out of plane S atoms vibrations, Figures 4 and 5; Figures S5 and S6, Supporting Information) around this pressure support the enhanced interlayer S‐S interactions. These pressure‐induced enhanced interlayer interactions together with robust intrinsic layer stackings prevent the layer sliding in 3R‐MoS_2_ (Figure 6; Figure S12, Supporting Information), in striking contrast to the layer sliding and corresponding first‐order phase transition in 2H‐MoS_2_.^[^ ^33^, ^46^ ^]^ Such a contrasting behavior between 2H‐ and 3R‐MoS_2_ polytypes, corresponds well to the layers stacking/ordering mechanism described by Katzke *et al.*,^[^ ^51^ ^]^ i.e. in the 3R stacking only the *R3m* space group is possible.

## 3 Conclusion

In summary, we investigated the structural, vibrational, and electrical transport properties of 3R‐MoS_2_ under pressure_._ We observed discontinuities in the plot of lattice parameters, *F‐f‐*plot, and Raman modes against pressure, which are evidence of the isostructural transitions. Metallization occurs because of the closure of the bandgap due to the reduction in the interlayer spacing and charge redistribution across vdW gap upon compression. This charge redistribution is a result of enhanced interlayer interactions which prevents the layer sliding in 3R‐MoS_2_. Pressure‐induced isostructural transitions and metallization in 3R‐MoS_2_ strikingly differ from the previous reports on 2H‐MoS_2_.^[^ ^32^, ^33^ ^]^ This study provides the first compelling evidence of pressure‐induced isostructural transitions and metallization in 3R‐polytypes of the TMDs, and will initiate further exploration of 3R‐polytypes of TMDs under pressure and enhance the possibilities of their use in the next‐generation device fabrication.

## 4 Experimental and Theoretical Methods

### 4.1 High‐Pressure Experiments

High pressure in situ Raman, synchrotron XRD and electrical measurements were performed using diamond anvil cells (DACs). More details about the experimental methods can be found in the Supporting Information.

### 4.2 Theoretical Calcuations

DFT‐based structural optimization, electronic band structure, and density of states (DOS) calculations of the 3R‐MoS_2_ were performed using the Vienna ab initio Simulation Package (VASP)^[^ ^36^ ^]^ utilizing the projector‐augmented‐plane wave (PAW)^[^ ^37^ ^]^ method. The exchange‐correlation function has been treated using both the Perdew‐Burke Ernzerhof for solids (PBEsol)^[^ ^38^ ^]^ and optPBE‐vdW^[^ ^39^ ^]^ methods within the generalized gradient approximation (GGA).^[^ ^40^ ^]^ For optimization, the planewave basis set was expanded with a kinetic energy cut‐off of 520 eV and a *k*‐point grid with a spacing of 0.2 Å^−1^. Calculations at 18, 20, 21, 22, and 45 GPa were performed using the PBEsol functional without including vdW forces, whereas calculations at 0, 20, 30, 31.5, 32, and 44.5 GPa were conducted using the optPBE‐vdW taking the vdW forces into consideration. *Ab‐initio* metadynamics simulations were performed to predict the structural evolution of *3R‐*MoS_2_ at 80 and 100 GPa. The kinetic energy cutoff used was 258.7 eV. Molecular dynamics (MD) simulations were performed using a MoS_2_ supercell containing 72 atoms. Each meta‐step consists of a first‐principles MD simulation in a canonical (NVT) ensemble. The metadynamics simulation used a Gaussian height of 225 *kbar* Å^3^and a width of (15 *kbar* Å^3^)^1/2^.

## Conflict of Interest

The authors declare no conflict of interest.

## Supporting information



Supporting Information

## Data Availability

The data that support the findings of this study are available from the corresponding author upon reasonable request.
