# Supplementary material for: Pressure‐Induced Metallization and Isostructural Transitions in 3R‐MoS2
Source: Adv Sci (Weinh). 2025 Jun 27;12(35):e05031. doi: 10.1002/advs.202505031 (PMC12463122; doi:10.1002/advs.202505031)
Supplement: Supplementary file 1 — Supporting Information [file ADVS-12-e05031-s001.docx]

**Supporting Information**

**For**

**Pressure-Induced Metallization and Isostructural Transitions in 3R-MoS_2_**

Azkar Saeed Ahmad,*^[a][b]^ Mangladeep Bhullar,^[c]^ Kenny Stahl,^[d]^ Wenting Lu,^[a]^ Taiyi Chen,^[a]^ Lei Feng,^[a]^ Xin Hu,^[a]^ Qian Zhang,^[a]^ Konstantin Glazyrin,^[e]^ Martin Kunz,^[f]^ Yusheng Zhao,^[g]^ Shanmin Wang,^[g]^ Yansun Yao,*^[c]^ and Elissaios Stavrou*^[a][b][h]^

[a] Dr. A. S. AHMAD, W. Lu, T. Chen, L. Feng, X. Hu, Q. Zhang, Prof. Dr. E. Stavrou

Materials Science and Engineering Department

Guangdong Technion-Israel Institute of Technology

Shantou 515063 (China)

Email: [azkar.ahmad@gtiit.edu.cn](mailto:azkar.ahmad@gtiit.edu.cn)

elissaios.stavrou@gtiit.edu.cn

[b] Dr. A. S. AHMAD, Prof. Dr. E. Stavrou

Guangdong Provincial Key Laboratory of Materials and Technologies for Energy Conversion (MATEC)

Guangdong Technion-Israel Institute of Technology

Shantou 515063 (China)

[c] M. Bhullar, Prof. Dr. Y. Yao

Department of Physics and Engineering Physics,

University of Saskatchewan

Saskatoon, Saskatchewan S7N 5E2 (Canada)

Email: [yansun.yao@usask.ca](mailto:yansun.yao@usask.ca)

[d] Prof. Dr. K. Stahl

Department of Chemistry

Technical University of Denmark

DK-2800 Lyngby (Denmark)

[e] Dr. K. Glazyrin

Deutsches Elektronen-Synchrotron (DESY)

D-22603 Hamburg (Germany)

[f] Dr. M. Kunz

Advanced Light Source

Lawrence Berkeley Laboratory

Berkeley, California 94720 (USA)

[g] Prof. Dr. Y. Zhao, Prof. Dr. S. Wang

Department of Physics

Southern University of Science and Technology

Shenzhen 518055 (China)

[h] Prof. Dr. E. Stavrou

Department of Materials Science and Engineering

Technion-Israel Institute of Technology

Haifa 32000 (Israel)

**Materials and Methods:**

**Sample synthesis and characterization.** Commercially available 2H-MoS_2_ (99.99 %) was used to study the phase transition between 2H and 3R polytypes under high P–T conditions. 3R-MoS_2_ was synthesized by high pressure-temperature solid state reaction in large volume cubic press. 2H-MoS_2_ was initially pressurized to 5 GPa followed by a heating cycle up 1800 ℃ for 15 Minutes. Temperature was measured by a chromel–alumel thermocouple. Prior to the experiments, 2H-MoS_2_ powder was compacted into a cylindrical pellet of 5 mm in diameter and 5 mm in height. An hBN capsule was used to contain the sample and to isolate the sample from thermocouple wires. In each experimental run, the sample was first compressed to a target pressure (5 GPa), and then heated to a desired temperature 1800 ℃ and soaked for 15 min before quenching and subsequent decompression to ambient conditions. The quenched specimen was initially allowed to cool down to RT and then decompressed to ambient conditions. At room temperature the purity and structure of the obtained 3R-MoS_2_ was confirmed by XRD (with a copper target, λ=1.540593 Å) and EDX as show in the Figure 1. The XRD data were analyzed using the Rietveld method in FullProf program.

**High-pressure XRD measurements.** Synchrotron radiation XRD experiments were performed in a Mao-Bell-type diamond anvil cell (DAC) with a culet 300 µm in diameter. The sample chamber was a hole of ∼100*μ*m diameter drilled in a preindented Re gasket. The specimen was loaded into the sample chamber, along with ruby as a pressure standard for calibration and Ne was used as pressure transmitting medium. Angle-dispersive XRD measurements were performed *in situ* under high pressure at the beamline P02.2, PETRAIII, Deutsches Elektronen-Synchrotron (DESY), Hamburg, Germany. For the data presented here, the energy of the synchrotron radiation was adjusted to 42.7 keV. Two-dimensional (2D) diffraction patterns were collected using a Perkin Elmer 1621 ScI-bonded amorphous silicon 2D detector (2048×2048 pixels, 200×200 µm pixel size) mounted orthogonal to the direction of the incident x-ray beam. A CeO_2_ standard (NIST 674B) was used to calibrate the sample-to-detector distance and the tilt of the detector relative to the beam path. The sample was exposed to an x-ray beam with a cross-section of 8 × 3 μm^2^ (FWHM). Second synchrotron XRD experiment was performed at Beamline 12.2.2, Advanced Light Source, Lawrence Berkeley National Laboratory. A Mars345 image plate detector was used. The wavelength of the X-ray was fixed to 0.4859 Å and the spot size of the X-ray beam was focused to about 10 x 10 *µ*m. The 2D patterns were integrated using the software package Dioptas.^[1]^

**High-pressure Raman spectroscopy measurements.** Raman spectroscopy experiments were performed in a Mao-Bell-type diamond anvil cell (DAC) with a culet 300 µm in diameter. The sample chamber was a hole of ∼100 μm diameter drilled in a preindented Re gasket. The specimen was loaded into the sample chamber, along with ruby as a pressure standard for calibration and He was used as pressure transmitting medium. Raman studies were performed using the 532 nm line of a solid-state laser for excitation in the backscattering geometry. The laser probing spot dimension was 4 µm. Raman spectra were recorded with a spectral resolution of 3 cm^−1^ using a single-stage grating spectrograph equipped with a CCD array detector. Ultra-low frequency solid-state notch filters allowed us to measure the Raman spectra down to 20 cm^−1^. The laser power was fixed to ~5 mW to get reasonably good signals without causing any laser/photo induced degradation of 3R-MoS_2_.

**Four probe electrical measurements.** High-pressure electrical resistance measurements for 3R-MoS_2_ were performed using the standard four-probe method. Mini-BX80 DAC with 400 μm culets was used for generating high pressure and T301 stainless steel preindented at ~23 GPa was used as a gasket and a hole with a diameter of 300 μm was drilled in the center of the indentation. Insulating *c*BN powder was used to fill the hole and cover the gasket to minimize the influence of the steel on resistance measurement. After compressing *c*BN to ~20 GPa a smaller hole of ~120 μm was drilled in the center and filled with 3R-MoS_2_ powder. Four electrodes were cut from Pt foil and used for the electrical connection with the specimen. To further ensure the insulation between steel and Pt leads, the gasket was covered with insulating paint. The sample and the Pt probes were in DAC to remove the spaces between powders for full contact of the sample with the probes. No pressure transmitting medium was used because it would permeate into the sample chamber and powder sample and cause the poor electrical connections between the sample and electrodes resulting in parasitic resistance. During resistance measurement and ruby powders were used as internal pressure calibrant.

**References:**

[1] C. Prescher, V. B. Prakapenka, DIOPTAS: A Program for Reduction of Two-Timensional X-ray Diffraction Data and Data Exploration *High Pressure Research* **2015**, *35*, 223-230.

**Figures:**


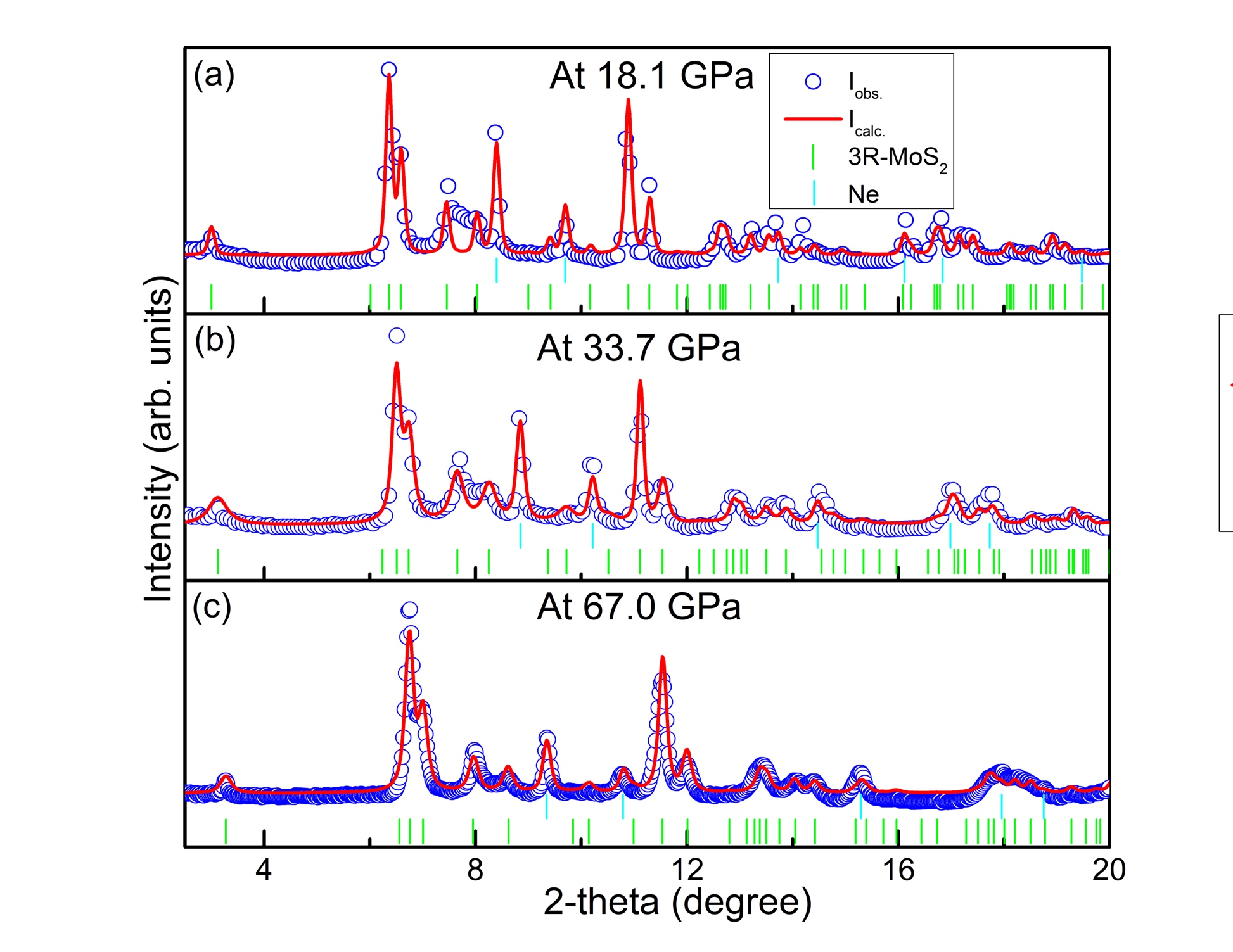


**Figure S1.** Representative Rietveld refinement results for 3R-MoS_2_ at 18.1 GPa (a), 33.7 GPa (b), and 67.0 GPa (c). The wavelength of the X-ray is 0.2904 Å.

**
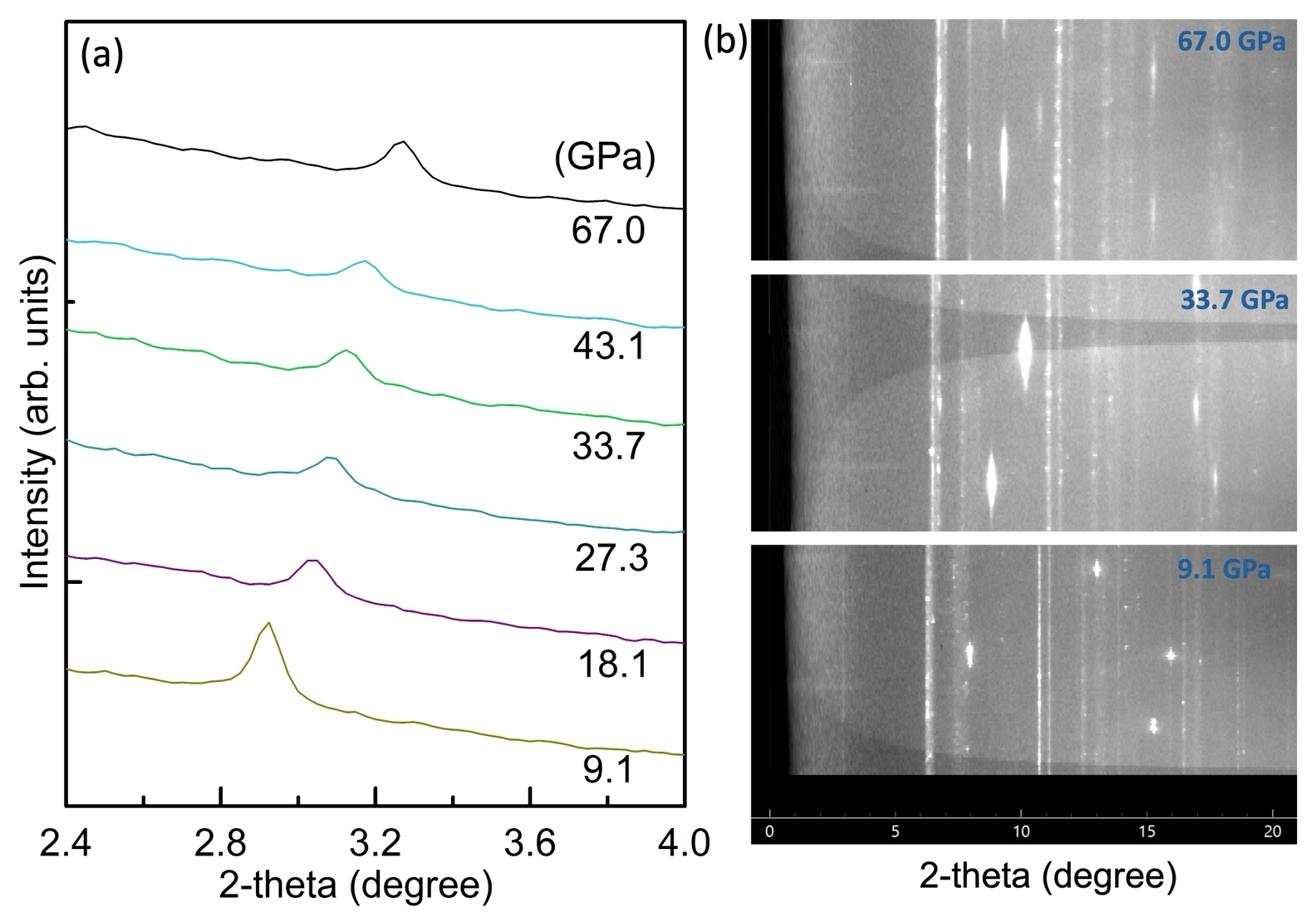
**

**Figure S2.** Zoom in view of first XRD peak around lower angles (i.e., ~3^0^) at selected pressures (a). Two dimensional XRD patterns of 3R-MoS_2_ at selected pressures (b).


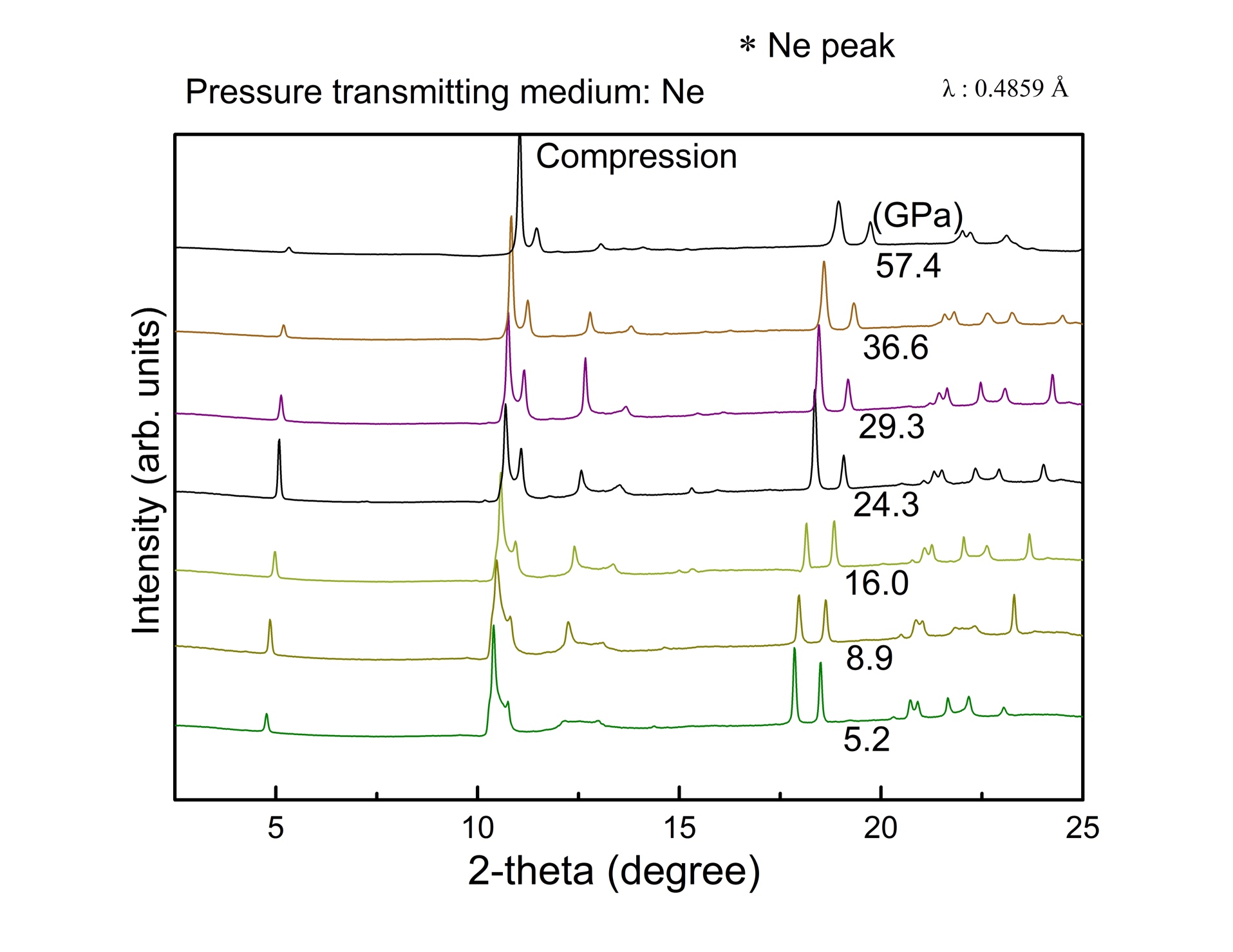


**Figure S3.** Representative synchrotron XRD patterns during compression of the second 3R-MoS_2_ sample. The wavelength of the X-ray was fixed to 0.4859 Å and the diffraction peaks from Ne (PTM) were masked.

**
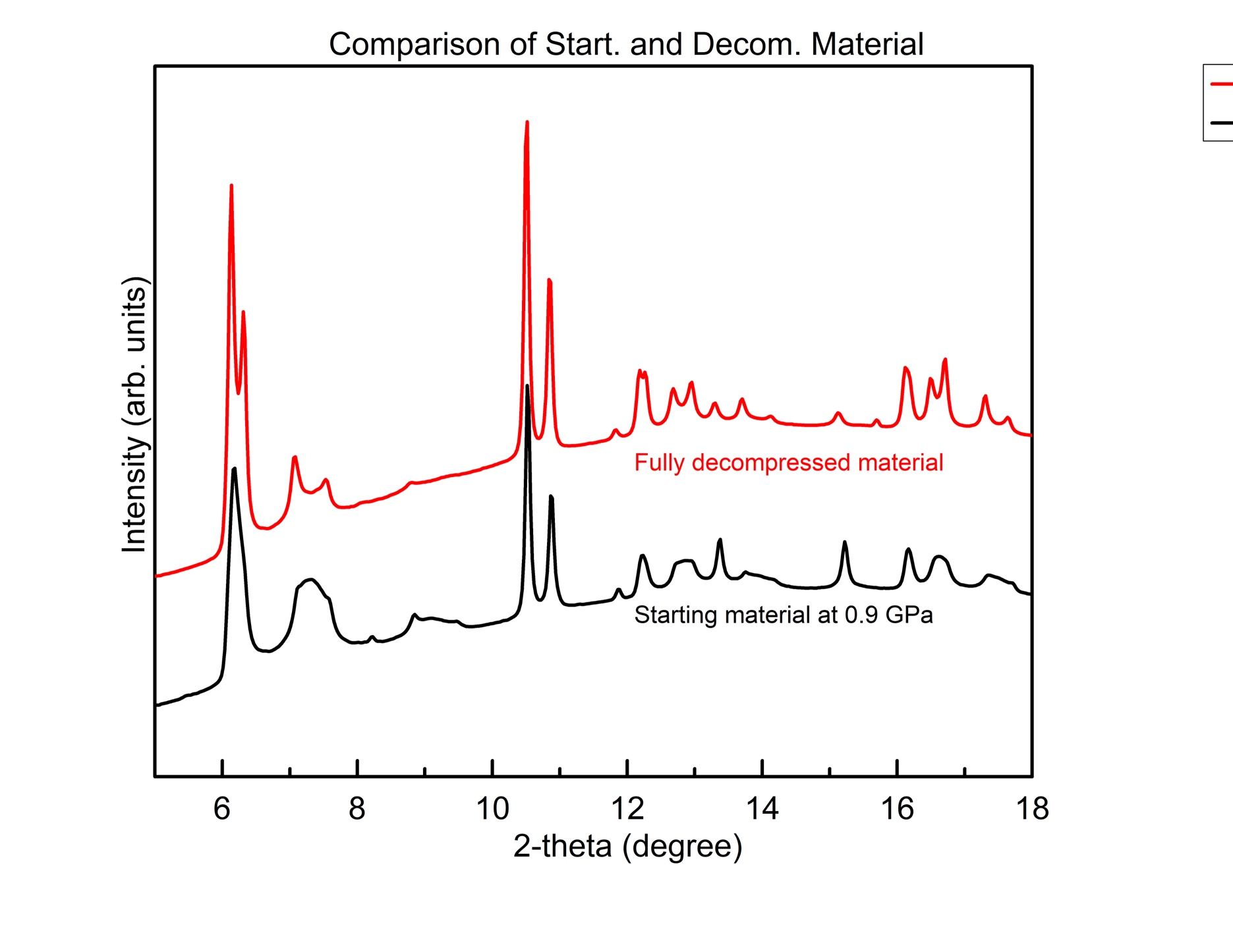
**

**Figure S4.** Comparison of starting and decompressed materials with synchrotron XRD measurements.


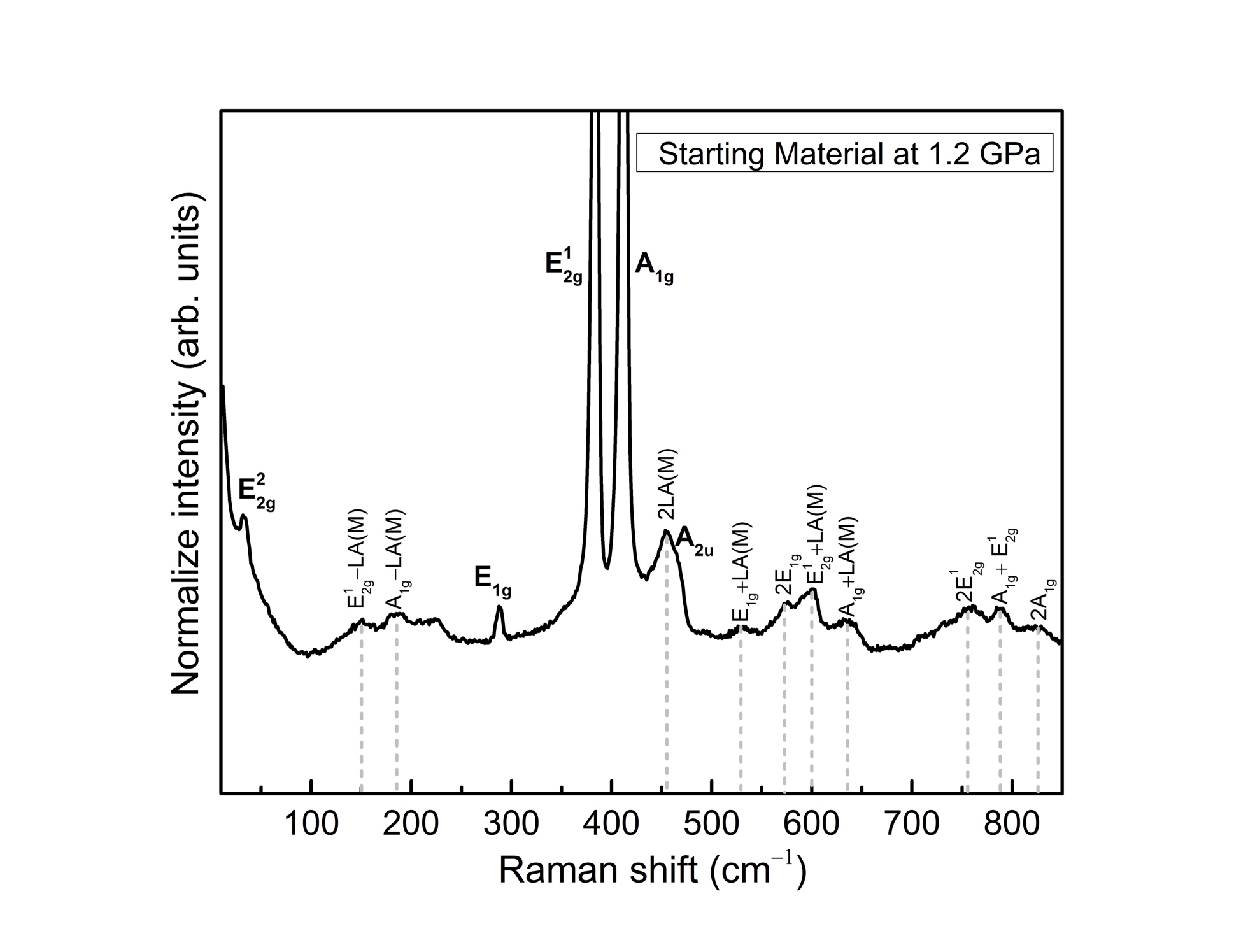


**Figure S5.** Zoom in view of Raman spectrum of 3R-MoS_2_ at 1.2 GPa during compression elaborating low intensity and 2^nd^ order modes.


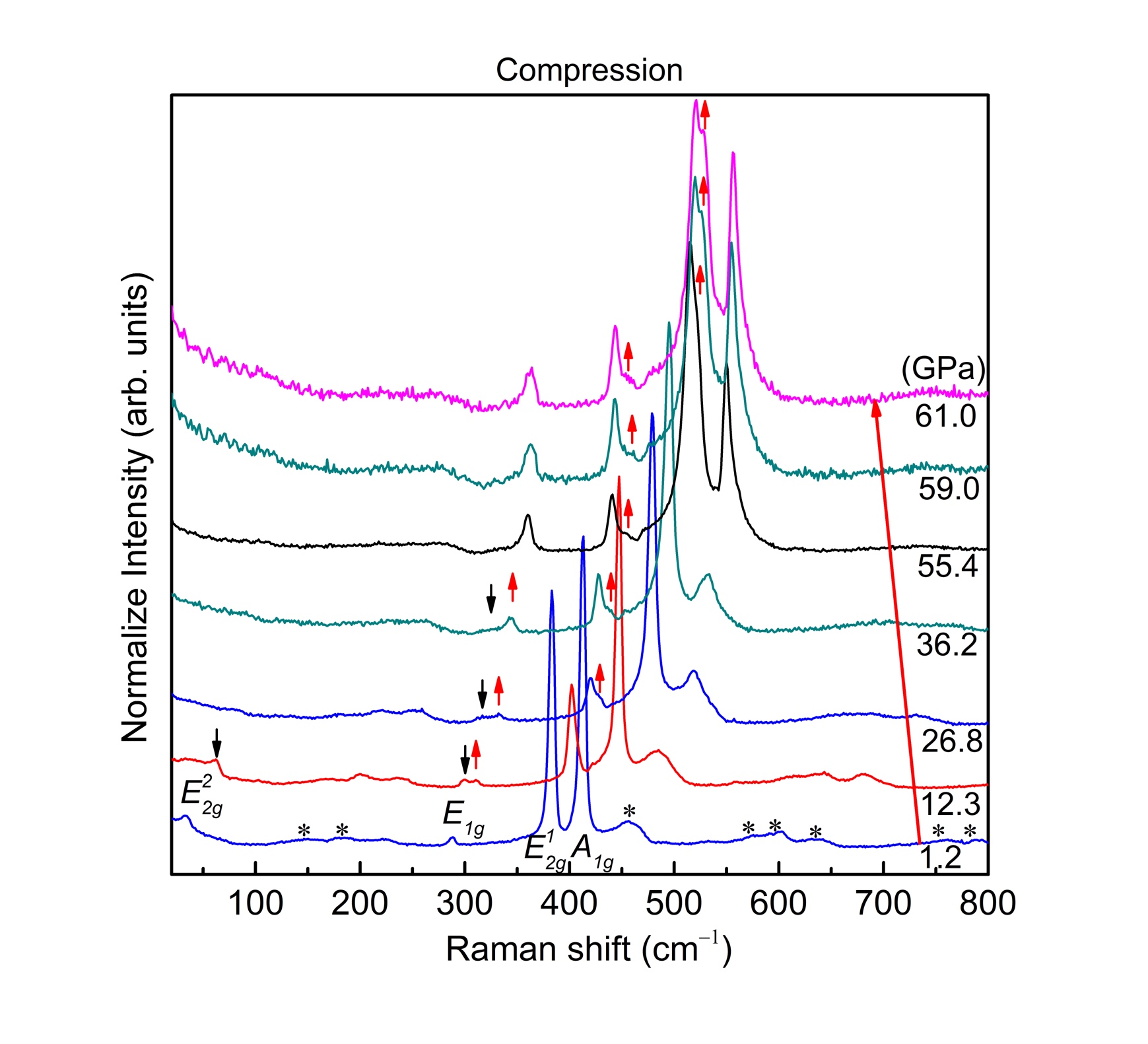


**Figure S6.** Raman spectra 3R-MoS_2_ at selected pressure points. The upward and downward arrows indicate the appearance/disappearance/splitting of phonon modes.


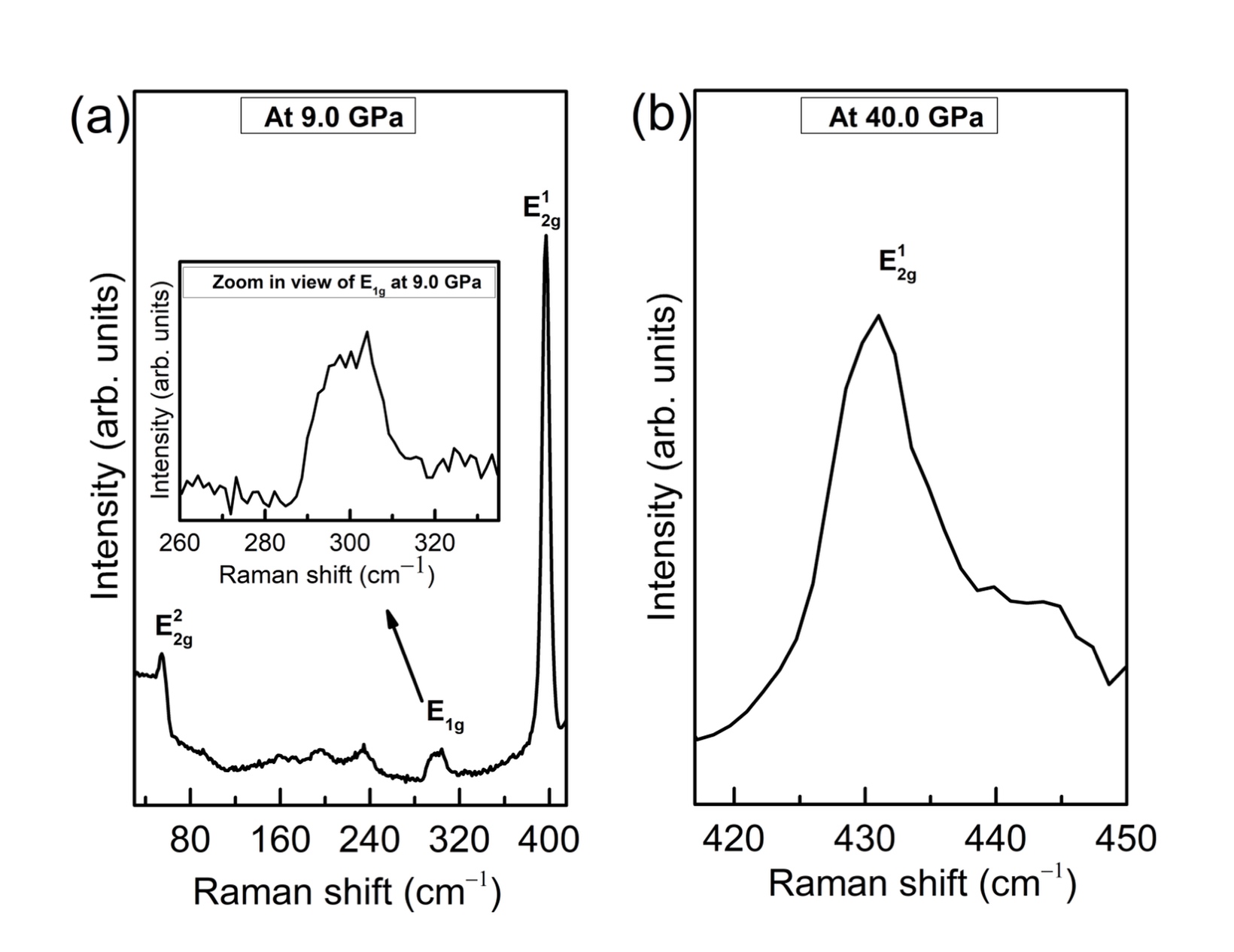


**Figure S7.** Raman spectra of 3R-MoS_2_ at selected pressure points illustrating the splitting of $E_{1g}$ at 9.0 GPa (a) and $E_{2g}^{1}$ at 40. 0 GPa.


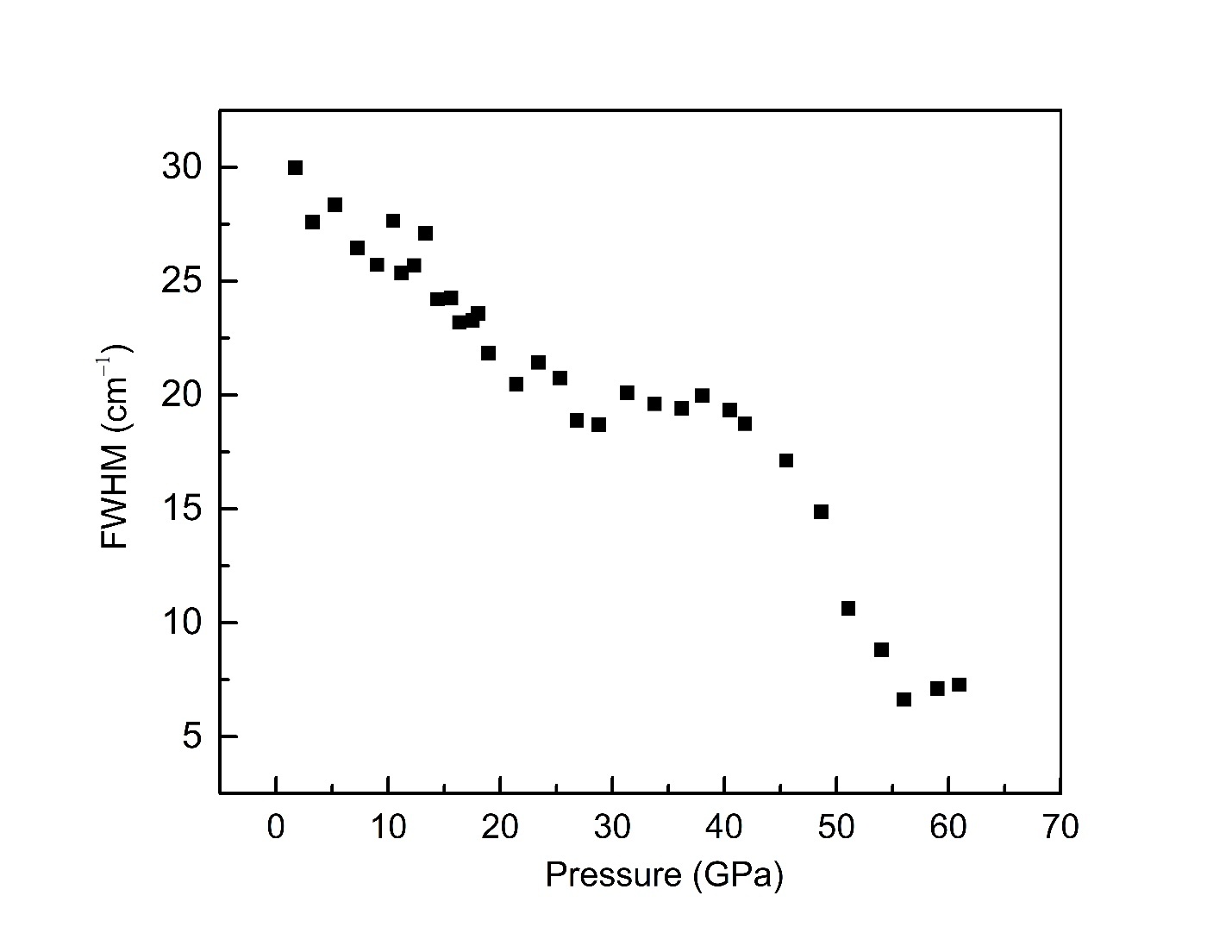


**Figure S8.** Plot of FWHM of 2LA(M)/A_2u_ mode against pressure.

**
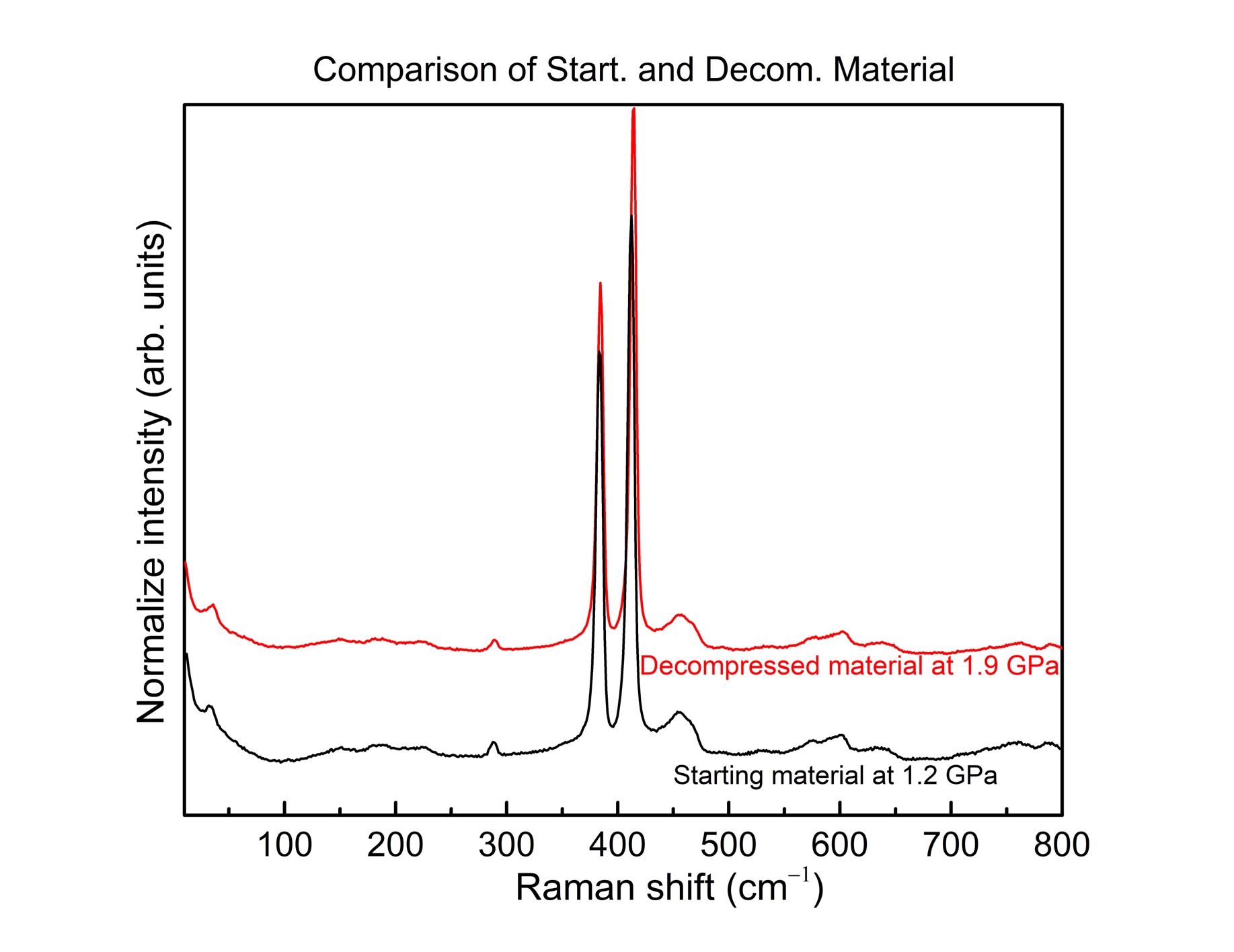
**

**Figure S9.** Comparison of starting and decompressed 3R-MoS_2_ from Raman spectroscopic measurements.


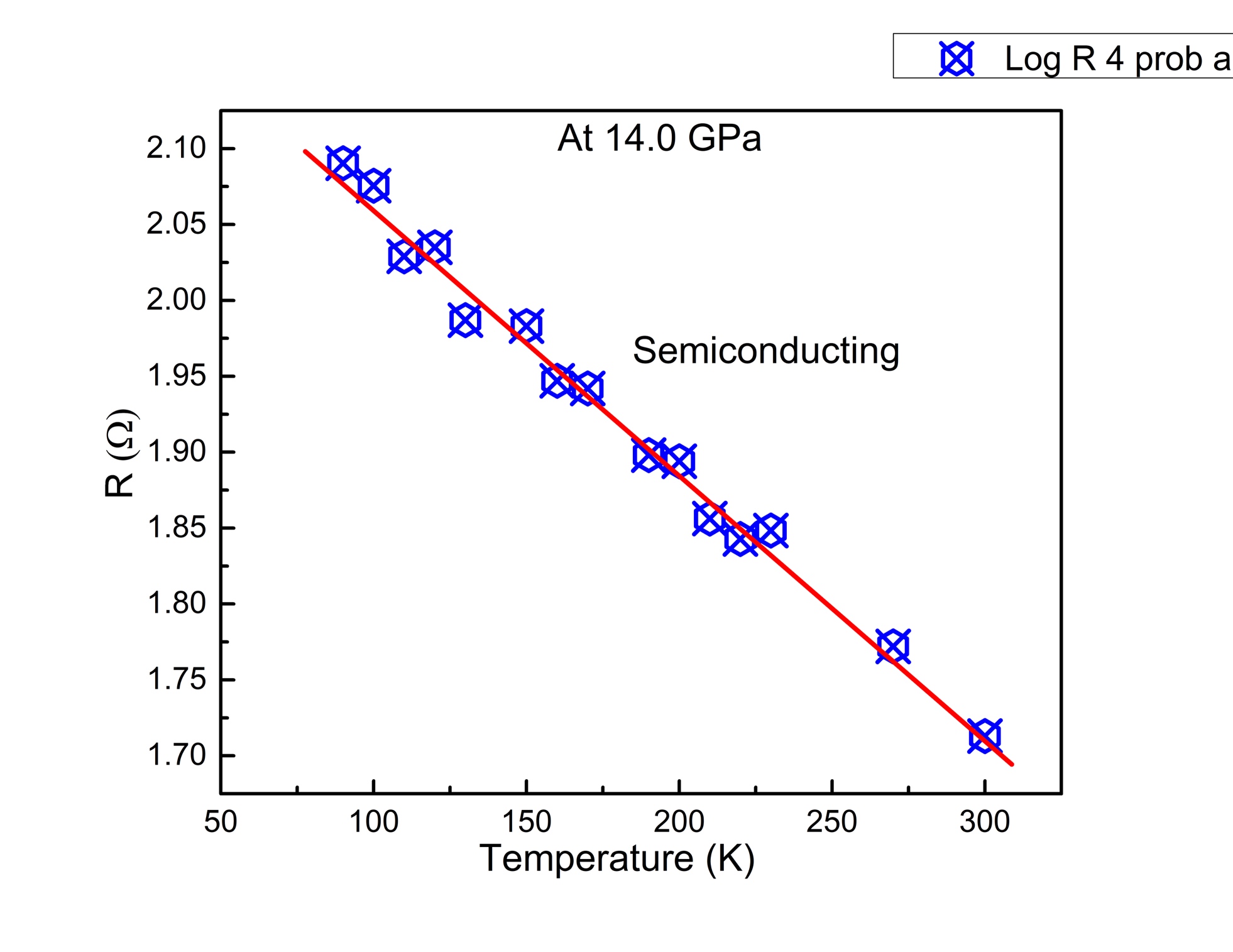


**Figure S10.** Temperature-dependence of electrical resistance at ~14.0 GPa. The red color solid line is just a guide to the eyes indicating semiconducting nature of 3R-MoS_2_ at ~14.0 GPa.


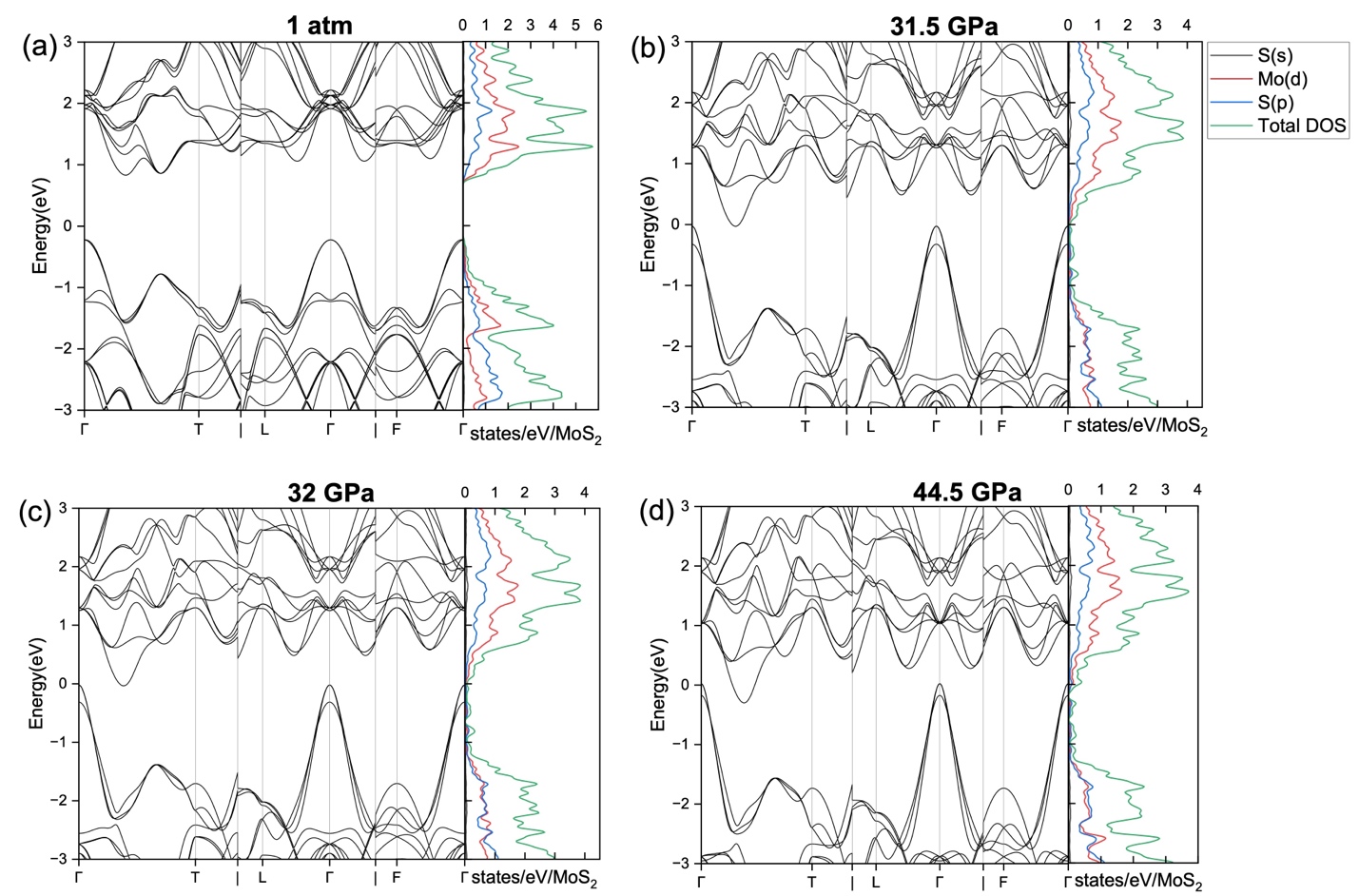


**Figure S11.** Band structure and DOS of 3R-MoS_2_ using optPBE-vdW at 0 GPa (a), 31.5 GPa (b), 32 GPa (c), and 44.5 GPa (d).


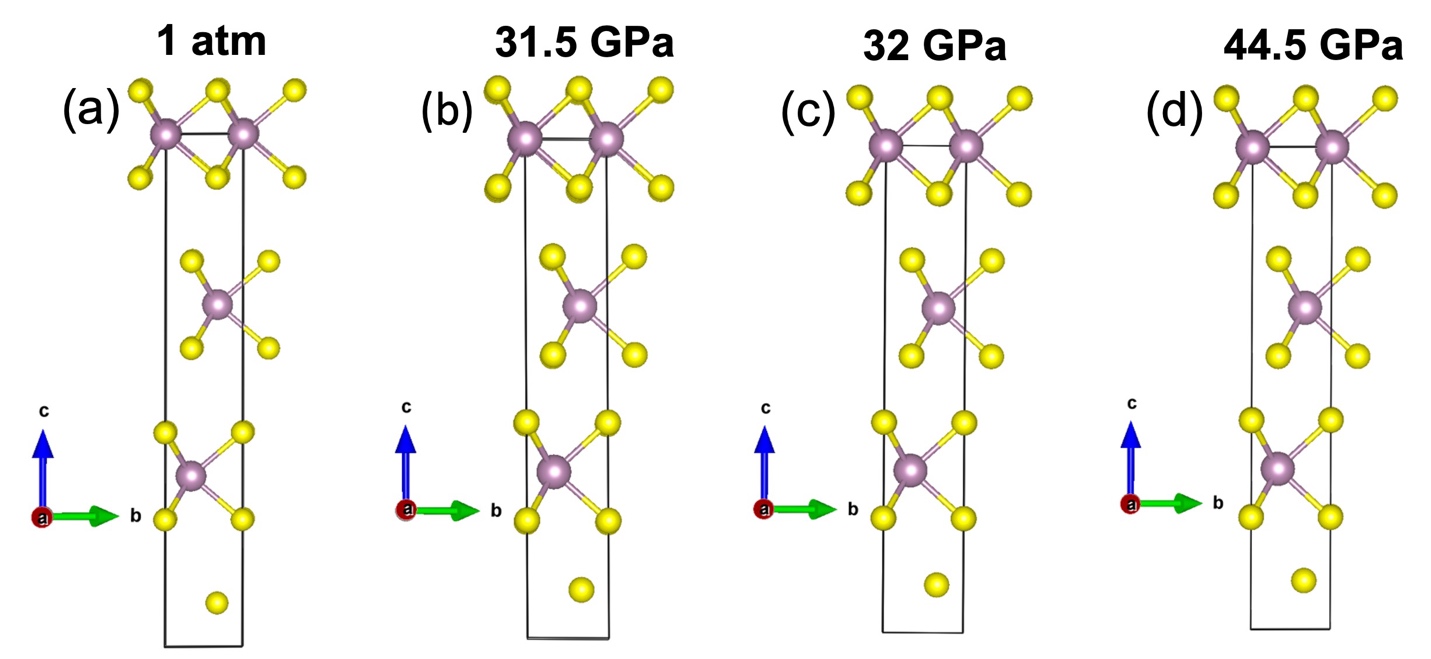


**Figure S12.** Illustration of the optimized structures in 3R-MoS_2_ using optPBE-vdW at 0 GPa (a), 31.5 GPa (b), 32 GPa (c), and 44.5 GPa (d). Big purple spheres represent Mo atoms and small yellow spheres represent S atoms.


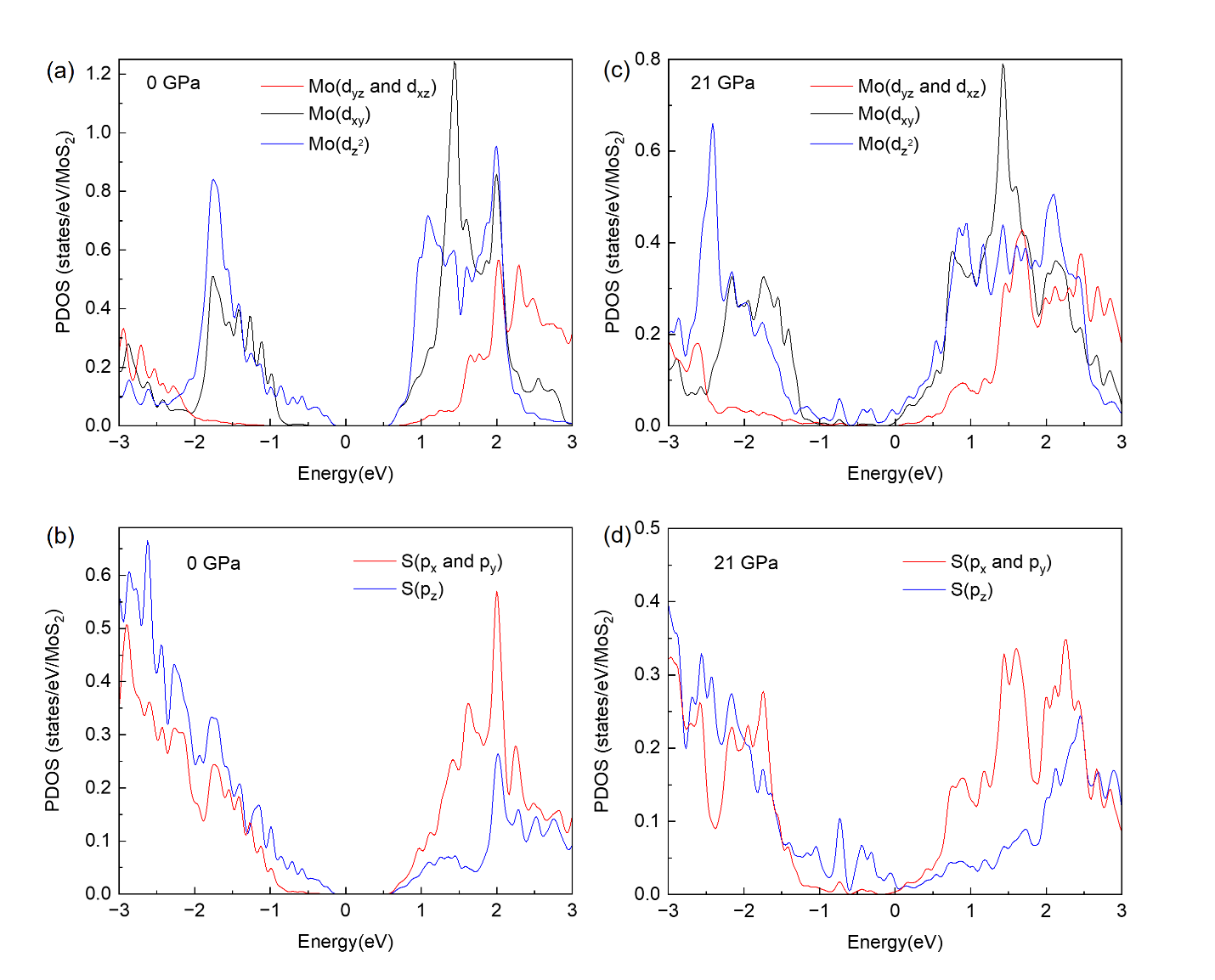


**Figure S13.** High-pressure **orbital** projected density of states (PDOS) of Mo and S in 3R-MoS_2_ at 0 GPa (a & b) and 21 GPa (c & d).

**Tables:**

**Table S1.** Refined structural parameters of as-synthesized 3R-MoS_2_ at ambient conditions. The Rietveld refinement was performed over the XRD pattern shown in the Fig. S1 (a).

| Space group | *R*3m (160) |
| --- | --- |
| Cell parameter *a* [Å] | 3.160 |
| Cell parameter *c* [Å] | 18.366 |
| Cell volume [Å^3^] | 158.825 |

**Table S2.** Bandgap values for 3R-MoS_2_ calculated under various pressure points using PBEsol and optPBE-vdW.

| **Pressure (GPa)** | **Bandgap (PBEsol)** |
| --- | --- |
| 0 | 0.9470 eV |
| 18 | 0.0831 eV |
| 20 | 0.0191 eV |
| 21 | -0.0218 eV (metallization) (Fig. 5) |
| 22 | -0.0523 eV |
| 45 | -0.7206 eV |
| **Pressure (GPa)** | **Bandgap (optPBE-vdW)** |
| 0 | 1.0517 eV (Fig. S9) |
| 20 | 0.3310 eV |
| 30 | 0.0529 eV |
| 31.5 | -0.0020 eV (semi-metallic) (Fig. S9) |
| 32 | -0.0144 eV (Fig. S9) |
| 44.5 | -0.3232 eV (Fig. S9) |
